# Exploring Wav2Vec2 XLSR-53 and Whisper-small for Telugu language speech-to-text: a fine-tuning approach

**DOI:** 10.3389/frai.2026.1883049

**Published:** 2026-09-09

**Authors:** Muzaffar Ahmad Dar, Sri Gani Kaarthikeya Kammula, Jagalingam Pushparaj, Venkata Sai Santhosh

**Affiliations:** 1 Department of Mathematics, School of Advanced Sciences, Vellore Institute of Technology, Vellore, Tamil Nadu, India; 2 Department of Quantum AI, School of Computer Science and Engineering, Vellore Institute of Technology, Katpadi, Vellore, Tamil Nadu, India; 3 Space Inventive, Hyderabad, Telangana, India

**Keywords:** fine-tuning, pre-trained models, speech-to-text, Telugu language, Wav2Vec2 XLSR-53, Whisper-small

## Abstract

Online voice-based applications and speech communication have grown as a result of the revolutionary rise of smart gadgets and social media. The rapid advancement of deep learning (DL) has transformed the field of audio processing, enabling smooth human-computer interaction. DL approaches have been used to develop speech-to-text (STT) systems across various languages and topics. These models require a large amount of training data: extensive corpora of continuous speech utterances collected from numerous speakers, along with their corresponding transcripts. In this study, we explore the use of state-of-the-art pre-trained models like Wav2Vec2 XLSR-53 and Whisper-small for developing STT systems in the Telugu language, addressing the challenge of limited data availability and demonstrating satisfactory results [23.62% Word Error Rate (WER), 4.12% Character Error Rate (CER)] even when fine-tuned on a smaller dataset. To evaluate model performance, we employed a k-fold cross-validation approach with values of *k* = 2 to *k* = 5, and compared the results with the conventional train-test split method. The results indicate that at *k* = 5, the Wav2Vec2 XLSR-53 model achieved a cross-validation Character Error Rate(WER) of 23.62% and a Character Error Rate (CER) of 4.12%, and the Whisper-small model yielded a cross-validation WER of 28.67% and a CER of 5.48%. These findings suggest that the k-fold cross-validation strategy, at k = 5, enhances the robustness of Wav2Vec2 XLSR-53 in low-resource language scenarios where training data is limited. For Whisper-small, however, the baseline train-test split (WER: 27.76%) outperformed all k-fold configurations tested (k = 2: 32.05%, k = 3: 29.72%, k = 4: 29.40%, k = 5: 28.67%), indicating that the benefit of k-fold cross-validation observed for Wav2Vec2 XLSR-53 does not generalize across model architectures. Additionally, the models were evaluated on unseen noisy data, and both models demonstrated satisfactory performance.

## 1 Introduction

Converting voice into text is known as speech-to-text, or STT. The requirement for STT in new languages and domains, the inherent diversity in speech, which is impacted by aspects like pitch, vocabulary, accents, and speaking rate, and the necessity for correct transcription make automatic speech recognition (ASR) a challenging endeavor, even though a lot of research has been done in recent decades (Ahmad Dar and Pushparaj, 2025). Virtual assistant technologies, human-machine interaction, voice-enabled chatbots, and voice-enabled equipment in the healthcare and banking industries, among many other applications, rely on STT systems (Pinto et al., 2022; Abdullah and Veisi, 2022). Many Indian languages do not have accurate STT systems, and therefore, as a result, research on the evolution of STT in different Indian languages is still quite popular.

Today's world is changing, and the technology being utilized is becoming advanced. Voice recognition technology is becoming a standard feature in various applications. According to (O'Shaughnessy 2008), one of the most sophisticated machine technologies for mimicking human behavior is STT. However, at present, STT systems are developed for languages with abundant annotated datasets, such as Hindi (Kumar and Mittal, 2021; Kumar and Aggarwal, 2020), English (Shi et al., 2021; Dar and Pushparaj, 2024; Song, 2019), Chinese (Yu et al., 2022), and Arabic (Alsayadi et al., 2021). Therefore, a language preservation mechanism is necessary to lessen the likelihood of language extinction. The process of conserving Telugu can affect the community in several ways, including raising public awareness of the language's extinction threat, which increases the desire of the general public to speak Telugu more frequently, encourages the younger generation to do the same, and boosts interest in learning the language.

Nearly 80 million people worldwide speak Telugu, a Dravidian language primarily spoken in southern India. After Tamil and Hindi, it is regarded as one of the most important languages in India and is the official language of both the states of Andhra Pradesh and Telangana (Reddy et al., 2025). However, the lack of large-scale datasets, pronunciation variations, and phonetic complexity makes it difficult to develop automatic STT systems for regional low-resource languages like Telugu.

STT encounters difficulties because of the inherent diversity in data for low-resource languages like Telugu. A system's performance can be impacted by variables including speaker changes, recurrent speech patterns, and differences in phoneme-grapheme alignment. A speaker may, for example, pronounce the same phrase on different occasions, creating inconsistencies that might affect a system's performance. We addressed this problem by fine-tuning the state-of-the-art pre-trained models Wav2Vec2 XLSR-53 and Whisper-small, which are already trained on large datasets (Pratap et al., 2020; Ardila et al., 2020; Salesky et al., 2021), to capture the complex internal features of the speech and accurately recognize them in the Telugu-annotated dataset. In this study, two well-known neural-based architectures—OpenAI's Whisper-small and Meta's AI Wav2Vec2 XLSR-53—are compared, and these frameworks were used to create an end-to-end STT system.

The contributions of the presented work are

- We investigate the effectiveness of Wav2Vec2 XLSR-53 and Whisper-small on the Telugu language, overcoming the scarcity of annotated data. Telugu was not among the languages used to pre-train Wav2Vec2 XLSR-53, and our results are therefore examined in the context of zero-shot cross-lingual transfer from phonologically related languages present in its pre-training data.
- We provide a systematic evaluation using both a traditional train-test split and 5-fold CV, offering model-specific insights into training consistency under limited annotated data.
- A hyperparameter analysis is conducted to identify effective training configurations for both models, fine-tuned on the OpenSLR Telugu dataset, demonstrating the adaptability of pre-trained models to regional, low-resource language tasks.
- The performance of the fine-tuned models is evaluated through assessment on noisy speech data and a qualitative error analysis comparing reference and predicted transcriptions to identify common recognition errors.

The remainder of this paper is organized as follows: Section 2 reviews related work. Section 3 details the methodology, including data preparation, model architecture, evaluation metrics, and experimental setup. Section 4 presents the experimental results, model evaluation, along with an in-depth discussion. Finally, Section 5 concludes the study and offers recommendations for future research.

## 2 Related studies

This section examines existing research concerning STT systems developed for Indian languages, placing particular emphasis on studies related to the Telugu language. In the past, STT systems for spoken Indian languages were developed through the integration of statistical approaches such as the Hidden Markov Model (HMM) and the Gaussian Markov Model (GMM). Among the reviewed literature, an early contribution in this field was reported by Saraswathi and Geetha (2004), who pursued isolated word recognition in the Tamil language through the application of an artificial neural network (ANN) framework. In Kaur and Singh (2016), the researchers focused individually on building a standardized corpus encompassing both text and speech in the Punjabi language and applied HMM for the recognition of Punjabi connected words, utilizing power-normalized cepstral coefficients as the primary feature set. Their system was evaluated across distinct acoustic conditions, yielding a WER of 16.28% in a clean audio setting and 32.08% under noisy circumstances. An ASR system was proposed for the Odia language employing the Kaldi toolkit, where acoustic modeling was conducted utilizing the HMM-GMM framework. The authors Karan et al. (2015), integrated both monophone and triphone modeling approaches for speech recognition tasks, with acoustic features processed using HMM-GMM techniques. Speech recordings were obtained from a total of 104 speakers, encompassing diverse age groups and dialectal variations, captured in mono-channel pulse code modulation format at a 16-bit resolution and sampled at 8 KHz. The training phase included 2130 spoken utterances, while evaluation was performed on 517 utterances from previously unseen speakers. The system reported a WER of 8.90% with monophone models and 1.74% using triphone configurations.

Over recent years, approaches based on deep learning (DL) have gained significant attention individually for advancing the development of STT systems across a range of Indian languages. A time-delay neural network architecture, multilingual in nature, is constructed wherein both acoustic modeling and language-specific attributes are jointly employed to perform decoding of test input sequences. Applying this methodology, resulting WERs achieved for the Gujarati and Tamil languages are 16.07% and 17.69%, respectively (Fathima et al., 2018). The researchers Islam et al. (2019) proposed and evaluated STT models for Bengali using two distinct approaches: CNN and RNN. The RNN model employed connectionist temporal classification (CTC) to enhance prediction performance. The CNN-based model achieved an accuracy of 86.058% for isolated speech-to-text conversion. The study Kumari et al. (2024) introduced an ASR framework for the Kannada language, designed as an end-to-end architecture grounded in deep learning. The proposed system integrates CNN alongside Bi-GRU, representing a specialized form of RNN. A total of 120 h of curated Kannada speech recordings were employed for model training. Two distinct deep learning configurations were examined experimentally. The result yielded a Character Error Rate (CER) of 18.62%. Experiments with a Kashmiri spoken numeral dataset of 7,200 spoken word samples representing various classes ranging from one to twenty. Each speech signal's Mel-spectrogram was first extracted, and the feature matrix that resulted was then formatted appropriately for the suggested model's input. The Kashmiri spoken numeral dataset yielded an average cross-validation accuracy of 85.28% when we applied a bidirectional long short-term memory (Bi-LSTM) network (Dar and Pushparaj, 2025). The proposed system incorporates a Bi-LSTM architecture, and the Seq2Seq model was trained utilizing a dataset spanning 21.63 h. This configuration yielded a WER of 36.48% and a CER of 15.69%. Augmentation data were integrated with the original baseline corpus, extending the total training duration to 31.84 h. Under this enhanced setting, the model demonstrated improved performance, achieving WER and CER values of 30.89% and 13.24%, respectively (Majhi and Saha, 2024). For the Tamil language, a framework has made use of publicly available NeMo resources. A language with limited resources can be studied using this method. This paper's goal is to reduce the WER statistic while transcribing speech-containing audio files, such as WAV files. The model achieved a WER of 17%, surpassing Google speech-to-text's 55% WER and Mozilla DeepSpeech architecture's 24.7% WER (Ghadekar et al., 2023).

The field of speech recognition systems exhibits only modest advancement at present concerning the Telugu language. A Telugu speech recognition and synthesis system has been introduced by the authors, utilizing a hybrid method that combines deep neural network (DNN) and HMM. This STT system is designed for effective recognition and synthesis of speech. The advancement in Telugu STT benefited from the multilingual dataset (Chadha et al., 2022), publicly introduced during the Interspeech 2021 challenge. The dataset contains six distinct languages, among which Telugu was included, and an STT proposed a baseline model for this dataset by employing the Wav2Vec2 XLSR-53 pre-trained framework, achieving a WER of 29.39%. The proposed (Krishna, 2021) framework integrates a single conformer-based encoder with a pair of transformer-based decoders: a phoneme decoder and a grapheme decoder, and experimental evaluation is carried out on a dataset released by Microsoft and SpeechOcean.com, covering Telugu, Tamil, and Gujarati with 40 h per language. For Telugu, the Conformer with dual grapheme and phoneme decoders achieves a WER of 25.28% and a CER of 6.18%.

A Telugu speech recognition and synthesis system has been introduced by the authors Reddy et al. (2025), utilizing a hybrid method that combines DNN-HMM. This STT system is designed for effective recognition and synthesis of speech. The presented architecture initiates by pre-processing the Telugu speech input, incorporating techniques such as silence elimination, resampling, and noise reduction. For feature extraction, Mel-Frequency Cepstral Coefficients (MFCCs) are utilized, followed by phoneme modeling based on HMM and classification via DNN to achieve reliable recognition. Authors Diwan et al. (2021) evaluated the Wav2Vec 2.0 framework through fine-tuning procedures applied across multiple Indian languages. Utilizing a dataset comprising 600 h of annotated speech, the study demonstrated a significant decline in error rates relative to conventional approaches. The author Chowdary et al. (2024) used the Whisper model and many speech performance indicators, such as WER, and built a multilingual ASR model for Dravidian languages, including Telugu. Using their basic configuration, the model achieved 61.2% WER for Telugu. Also, the authors Jain and Bhowmick (2025) developed an STT framework for the Telugu language; the pretrained transformer models Whisper-small and Wav2Vec2-XLSR-53 were independently fine-tuned. Evaluation was conducted using CER along with WER. Wav2Vec2 XLSR-53 demonstrated a WER value of 28.67% and a CER of 4.92%, whereas Whisper-small recorded a WER of 30.04%, and a CER of 10.31%, thereby indicating the superior effectiveness of Wav2Vec2 XLSR-53 over Whisper-small.

As mentioned above, the techniques exhibit greater efficiency; their application demands extensive volumes of speech data for both training and evaluation phases. However, the limited availability of annotated speech resources poses a significant challenge for numerous Indian languages, such as Telugu. To address the scarcity of labeled Telugu speech data, this study employed a k-fold cross-validation strategy to manage limited-duration datasets. Fine-tuning was performed using a small set of annotated samples on pre-trained Wav2Vec2 XLSR-53 and Whisper-small models, resulting in improved CER and WER. This research concentrates on constructing an STT system tailored for the Telugu language by leveraging the capabilities offered through self-supervised learning (SSL) approaches.

## 3 Methodology

**Figure 1:**
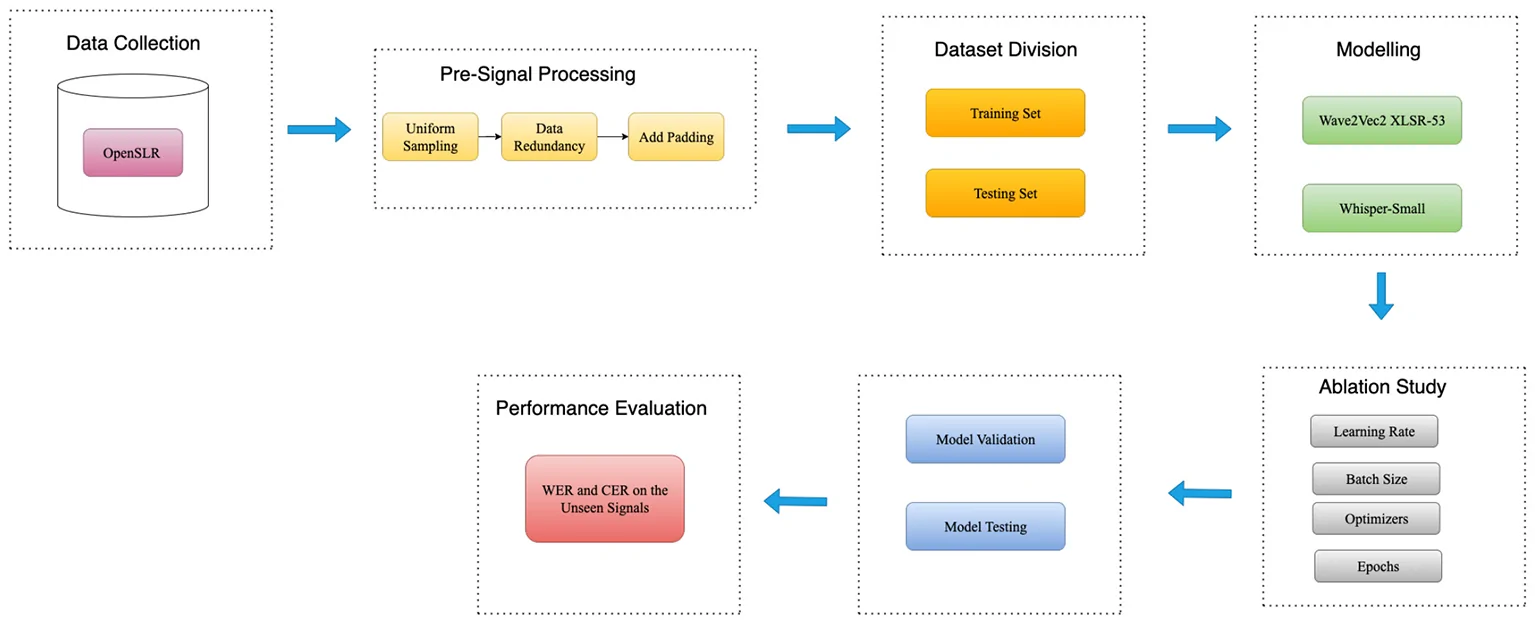
Overall methodology for the proposed work.

In this section, we provide the detailed methodology that we adopted during our approach. The frameworks employed are Wav2Vec2 XLSR-53 (Baevski et al., 2020) and Whisper-small (Radford et al., 2022). These models have been employed in diverse applications, such as STT (Abdullah et al., 2024) and speaker diarization (Abdullah et al., 2025). Their training process involves two distinct phases: fine-tuning and pre-training. During the pretraining phase, speech audio is transformed into a latent space where segments are masked, and a contrastive learning objective is formulated. This objective leverages predictions generated by the transformer and utilizes quantized latent speech representations, thereby facilitating the acquisition of rich contextual features solely from raw speech signals. The process advances to the fine-tuning stage, which utilizes annotated data for task-specific adaptation. The overall methodology of this work is illustrated in Figure 1.

### 3.1 Dataset description

The process of acquiring data of low-resource linguistic domains, such as Telugu, involves notable obstacles stemming from the limited availability of speech corpora that are annotated. To overcome this constraint, a publicly accessible dataset—namely, the OpenSLR66 (He et al., 2020) was employed. It comprises nearly 6 h worth of recorded speech encompassing varied voice types, dialectal diversity, and accent variations, with contributions equally balanced between male and female speakers. This resource is further enriched with hand-aligned transcription annotations, thereby facilitating the use of SSL approaches. In addition, to gain further insight into the corpus's lexical properties. The dataset employed in this study comprises a total of 3,558 training sentences and 890 testing sentences, contributing to a corpus of 22,430 words, of which 7,935 are unique. The phonetic inventory includes 45 distinct phonemes. The recordings were collected from 23 to 24 speakers, ensuring a balanced gender representation across male and female participants.

### 3.2 Data pre-processing

Preprocessing represents a fundamental aspect in dataset preparation aimed at STT systems, contributing to both model efficiency and accuracy throughout the phases of training as well as evaluation. A customized sequence of preprocessing operations was applied to each dataset to improve its practical utility. For the OpenSLR corpus, the procedure included bringing audio clips to a standardized loudness threshold, converting all sound samples to a fixed sampling frequency of 16 kHz, and removing segments characterized by irrelevant silence. Furthermore, textual data underwent refinement through the elimination of superfluous characters, thereby supporting effective token segmentation and downstream linguistic processing. These methodological steps facilitated conformance to established input data standards, enhancing STT model integration.

In the case of the Wav2Vec2 XLSR-53 framework, the preprocessing extended its scope past routine normalization and transcript sanitation. Linguistic transcripts were transformed into character-level (grapheme) representations and adjusted to fixed-length sequences using padding techniques, promoting efficient temporal sequence handling for CTC-based training. This design preserved Telugu language phonetic intricacies, enabling accurate phonological representation. Conversely, the Whisper-small architecture employed a minimalistic preprocessing pipeline by utilizing unprocessed audio and corresponding transcription content, foregoing explicit token segmentation and padding operations. Instead, Whisper's internal adaptation mechanisms accommodated varying input durations, thereby lowering preprocessing complexity. To uphold the accuracy of data associations, a supplementary validation process was added to confirm audio-text correspondence and prevent transcription inconsistencies. The datasets were meticulously adapted for robust STT model training, improving recognition and transcription accuracy for Telugu speech data by resolving these preprocessing intricacies.

### 3.3 Experimental setup for cross validation

K-fold cross-validation is a standard technique; we use this in our study because of the low-resource nature of Telugu ASR. When the model is trained using the traditional train-test split. The model learns from a single training dataset that is randomly selected, and its performance is dependent on the specific training data. In contrast, k-fold cross-validation allows us to maximize data utilization by ensuring that all the data is partitioned into subsets, and each subset serves as both training and validation, thereby providing a more robust estimate of model generalization.

For STT tasks in low-resource languages, the advantage of cross-validation training is that it provides a robust model capable of generalizing to unknown patterns, thereby mitigating the risk of overfitting and reducing bias introduced by small datasets. A 5-fold cross-validation procedure (*k* = 5) was employed to evaluate the performance of the models. For each fold, the model was trained on a randomly assigned training subset corresponding to that fold. For each subsequent value of *k*, one fold was reserved for validation and the remaining folds were used for training. For example, when *k* = 3, the dataset is partitioned into three distinct subsets (fold-1, fold-2, and fold-3). In the first iteration, fold-1 and fold-2 are employed for training, while fold-3 is reserved for validation. In the second iteration, fold-2 and fold-3 serve as the training sets, and fold-1 is used for validation. Finally, in the third iteration, fold-1 and fold-3 are utilized for training, with fold-2 designated for validation. The data distribution for each of *k* = 2, 3, 4, 5 is distinct; therefore, in the case of *k* = 5, the combination of its fold-1, fold-2, fold-3, fold-4, and fold-5 constitutes the complete 5-fold ensemble.

#### 3.3.1 Relationship between cross-validation folds and the held-out test set

The dataset was first partitioned into a fixed training set (3,558 sentences) and a fixed test set (890 sentences) via a single stratified split. This 890-sentence test set remains constant and is never included in, or rotated through, the k-fold partitions described above. The k-fold cross-validation procedure operates on the 3,558-sentence training set, generating rotating training/validation folds for model training and validation. The fixed test set is used only once, after k-fold-based training is complete, to obtain the final held-out performance figures. To avoid ambiguity, we distinguish these two quantities throughout the manuscript as *k-fold Validation set* (computed on the rotating in-training-set validation folds) and *Held-out test set* (computed on the fixed 890-sentence test set).

#### 3.3.2 Speaker-independence across folds

Fold partitioning was performed using standard utterance-level k-fold splitting (KFold, scikit-learn), which assigns individual utterances to folds without accounting for speaker identity. Given that the underlying Telugu speech corpus contains only 23–24 speakers, utterances from the same speaker may appear in both the training and validation partitions within a given fold. As a result, the reported k-fold validation set values should be interpreted as an estimate of performance under this speaker-overlapping regime, rather than a strict measure of robustness to entirely unseen speakers.

### 3.4 Wav2Vec2 XLSR-53 model description

This study explores the efficacy of adapting the Wav2Vec2 XLSR-53 (Conneau et al., 2020) architecture developed by the Meta AI Research group. This model, pre-trained across a set of 53 languages, stands at the forefront of methodologies aimed at extracting meaningful textual representations directly from unprocessed audio signals. Its design comprises the following core components:

1. Feature Encoder: Upon receiving a matrix of raw audio, a stack of CNN layers produces temporally aligned latent speech representations across *T* discrete intervals, compressing the raw waveform into a denser sequence of feature vectors without manual feature engineering.
2. Transformer Encoder (Context Network): These latent representations are processed through a stack of transformer layers, which derive contextually rich outputs at each of the *T* time points by allowing each frame to attend to surrounding frames, thereby capturing phonetic patterns and longer-range speech context.
3. Quantization Module: During self-supervised pre-training only, a separate branch discretizes the continuous latent representations into a fixed set of codebook entries, summarizing continuous audio into a finite vocabulary of discrete speech units. This module operates during pre-training and is not used during downstream fine-tuning.
4. Contrastive Pre-training Objective: During pre-training, a subset of CNN output frames is masked, and the model is trained to correctly identify the true quantized representation of each masked frame from among a set of distractors. This objective enables the model to learn general-purpose audio representations without requiring transcribed text.
5. CTC Head (Fine-tuning): As the pre-trained model produces only latent audio representations and does not itself output text, a linear classification layer is added on top of the transformer encoder during fine-tuning. This layer maps encoder outputs to characters (graphemes) and is trained using CTC loss (Graves et al., 2006) to align audio frames with the corresponding transcript characters.

In STT systems, establishing accurate alignments between individual characters and their corresponding positions within continuous audio streams is a known challenge. The CTC loss computes the divergence between the unsegmented acoustic signal and a target sequence label consisting of characters by summing the probability distribution over all feasible alignment paths. This process produces a differentiable loss value, enabling gradient-based learning for each model input. The alignment paradigm assumes a many-to-one correspondence, thus requiring the output sequence to align in length with the input feature sequence. Adapting the model to the Telugu language via targeted fine-tuning enhances model efficacy. We assessed these fine-tuned versions using conventional ASR metrics: the WER and CER. These measures evaluate the model's capacity to yield accurate speech transcriptions, capture the intended content of the input audio, and maintain generalization across unseen utterances.

### 3.5 Whisper-small model description

In recent developments, OpenAI introduced Whisper (Radford et al., 2022), an all-purpose model for speech recognition, which has been trained utilizing a wide range of audio samples. This multitasking architecture demonstrates capabilities in recognizing speech across languages, performing translations, and identifying languages. Whisper-small, comprising more than 244 million trainable parameters, stands in contrast to Wav2Vec2, which consists of approximately 316 million parameters.

Nonetheless, two principal distinctions should be noted when drawing comparisons between Wav2Vec2 and Whisper: (1) Whisper computes the WER following a specialized text normalization process that precedes the WER assessment. This preprocessing step enhances output fidelity by aligning transcriptions with reference text and mitigating frequent transcriptional inaccuracies. (2) Despite its benefits, this strategy might not accurately simulate practical conditions, where inconsistencies and noise in training corpora are commonplace. For instance, datasets may contain silent segments incorrectly annotated with complete sentences.

For our experimental framework, the model Whisper-small was chosen as the foundational component. This ASR model, designed for multiple languages, is a pre-trained architecture encompassing approximately 242 million individual parameters and supports 99 language variants. Structurally, Whisper is based on a transformer architecture utilizing encoder-decoder mechanisms, where the log-mel spectrogram derived from the input speech is processed through the encoder. Simultaneously, the decoder, initiated with designated start tokens, integrates speech-derived information into its Transformer layers via cross-attention. The decoder generates the next token in the sequence through autoregressive prediction. By utilizing this framework and adhering to a defined format, speech-based in-context learning can be conducted with the pre-trained Whisper model.

### 3.6 Pre-training

In this study, we conduct experiments involving two distinct pretrained models. The first, referred to as Whisper-small (Radford et al., 2022), incorporates a base architecture consisting of approx. 244 million parameters and has been trained utilizing approx. 680,000 h of weakly supervised, multilingual, and multitask audio data. The second model employed in our experimentation is Wav2Vec2 XLSR-53 (Conneau et al., 2020), which adopts a large architecture with approx. 316 million parameters and has undergone training on 56k h of speech drawn from 53 languages.

### 3.7 Fine-tuning

The process involves taking pretrained architectures and fine-tuning them by adding a fully connected module atop the context-processing network. The output dimension of this added layer corresponds to the specific task's vocabulary size. For optimization, a CTC loss (Graves et al., 2006) function is employed. Fine-tuning procedures are conducted individually on each of the pretrained models. For Wav2Vec2 XLSR-53, the convolutional feature encoder was frozen during fine-tuning, while the transformer encoder layers and the newly added CTC head were trained. The vocabulary was built from the Telugu training transcripts at the character level. For Whisper-small, we used the full pre-trained tokenizer/vocabulary (no custom Telugu vocabulary was built), and fine-tuning was performed on the entire encoder-decoder stack with the language = “Telugu,” task = “transcribe” setting—no layers were frozen.

### 3.8 Evaluation metrics

Two principal metrics were employed to evaluate the performance of the pre-trained Wav2Vec2 XLSR-53 and Whisper-small models: CER and WER. In the context of STT, WER measures performance by quantifying word-level inaccuracies, whereas CER offers a finer perspective by calculating recognition errors at the level of individual characters. Through the application of both CER and WER, a comprehensive analysis of the model's effectiveness was facilitated. The standard evaluation of ASR accuracy involves WER and CER. WER is determined by calculating the proportion of cumulative errors, including insertions, deletions, and substitutions, against the number of words in the reference transcript. Analogously, CER quantifies character-level deviations. The mathematical equations for CER and WER are given below.


$$\begin{array}{l} \text{CER}=\frac{S_{\text{Subs}}+I_{\text{Ins}}+D_{\text{Del}}}{\text{Total Characters in Reference Text}} \end{array}$$ (1)

$$\begin{array}{l} \text{WER}=\frac{S_{\text{Subs}}+I_{\text{Ins}}+D_{\text{Del}}}{\text{Total Words in Reference Text}} \end{array}$$ (2)

where

- *S*_Subs_: words or characters that had been identified as incorrectly recognized,
- *D*_Del_: words or characters that the system missed in recognition.
- *I*_Ins_: words or characters that have been added by the model

## 4 Results and discussions

This section will give the detailed results on the 5-fold validation for both Wav2Vec2 XLSR-53 and Whisper-small. It provides an overview of details concerning the parameters used during training for both models. It also presents an ablation analysis involving the application of varying cross-fold validation approaches. Additionally, a detailed discussion is offered regarding findings drawn from comprehensive experiments conducted for STT tasks in Telugu. The experimental results are reported with the objective of evaluating how different cross-folds influence performance on the Telugu STT task.

### 4.1 Selection of training parameters

In this section, we provide a detailed discussion of the parameters employed for fine-tuning the state-of-the-art models, including Wav2Vec2 XLSR-53 and Whisper-small. The subsequent sections offer an in-depth analysis of the selected hyperparameters. The performance of both models was evaluated using WER and CER metrics. The selection of hyperparameters for model fine-tuning was performed prior to conducting k-fold cross-validation. This approach was adopted to reduce the substantial computational cost and training time associated with repeatedly performing hyperparameter optimization within each fold for large pre-trained models.

#### 4.1.1 Selection of the learning rate

To identify the optimal learning rate for fine-tuning, a series of experiments was conducted, shown in Table 1. Among the tested configurations, the learning rate of 3e-4 yielded the best results for the Wav2Vec2 XLSR-53 model, achieving a WER of 38.13% and a CER of 9.39%. In contrast, the optimal performance for the Whisper-small model was observed at a learning rate of 1e-5, which resulted in a WER of 40.87% and a CER of 10.37%.

**Table 1:** Ablation study for Wav2Vec2 XLSR-53 and Whisper-small based on learning rate.

Wav2Vec2XLSR-53		
Learning rate	WER	CER
**3e-4**	**38.13%**	**9.39%**
2e-4	38.77%	9.65%
1e-4	41.93%	10.25%
**Whisper-small**		
Learning rate	WER	CER
**1e-5**	**40.87%**	**10.37%**
1e-4	51.67%	15.67%
1e-3	41.26%	10.86%

Bold values indicate the best performance.

#### 4.1.2 Selection of the batch size

An ablation study was conducted to examine the effect of varying batch sizes on the performance of the Wav2Vec2 XLSR-53 and Whisper-small models as shown in Table 2. Batch sizes of 16, 32, and 64 were tested, and the results indicated that a batch size of 16 produced the most favorable outcomes for both models. The Wav2Vec2 XLSR-53 achieved a WER of 36.87% and a CER of 9.17%, while the Whisper-small model obtained a WER of 36.79% and a CER of 9.92%.

**Table 2:** Ablation study for Wav2Vec2 XLSR-53 and Whisper-small based on batch size.

Wav2Vec2 XLSR-53		
Batch Size	WER	CER
**16**	**36.87%**	**9.17%**
32	38.13%	9.28%
64	46.88%	11.34%
**Whisper-small**		
Batch Size	WER	CER
**16**	**36.79%**	**9.92%**
32	40.87%	10.37%
64	64.87%	16.80%

Bold values indicate the best performance.

#### 4.1.3 Selection of the optimizer

To assess the influence of the optimization algorithm on model performance, we evaluated the Adam optimizer during experimentation. Table 3 reports the final performance achieved using the Adam optimizer for both models. For the Wav2Vec2 XLSR-53 model, the Adam optimizer resulted in a WER of 36.87% and a CER of 9.17%. Similarly, the Whisper-small model fine-tuned with the Adam optimizer achieved a WER of 36.79% and a CER of 9.92%.

**Table 3:** Performance of Wav2Vec2 XLSR-53 and Whisper-small using the Adam optimizer.

Model	WER	CER
Wav2Vec2 XLSR-53	36.87%	9.17%
Whisper-small	36.79%	9.92%

#### 4.1.4 Selection of the number of epochs

The impact of the number of training epochs on model performance was investigated by conducting experiments with 20, 30, and 45 epochs, as shown in Table 4. The Wav2Vec2 XLSR-53 model demonstrated optimal performance at 45 epochs, achieving a WER of 34.04% and a CER of 8.75%. In contrast, the Whisper-small model exhibited its best results at 30 epochs, with a WER of 34.79% and a CER of 9.44%.

**Table 4:** Ablation study for Wav2Vec2 XLSR-53 and Whisper-small based on training epochs (20, 30, 45).

Wav2Vec2 XLSR-53		
Epochs	WER	CER
20	36.87%	9.17%
30	35.33%	9.00%
**45**	**34.04%**	**8.75%**
**Whisper-small**		
Epochs	WER	CER
20	36.79%	9.92%
**30**	**34.79%**	**9.44%**
45	36.80%	9.87%

Bold values indicate the best performance.

### 4.2 Analysis on k-fold validation of Wav2Vec2 XLSR-53

This section presents a comprehensive breakdown of outcomes acquired utilizing the Wav2Vec2 XLSR-53 model, achieved through implementing 5-fold cross-validation. Additionally, it outlines the individual performances corresponding to folds k=2, 3, 4, and 5, thereby offering insights into the functioning of the Wav2Vec2 XLSR-53 for the particular language.

Table 5 illustrates how the Wav2Vec2 XLSR-53 architecture performs in WER, CER, and evaluation loss across various k-fold validation schemes. In this context, the label “Base” model signifies that a conventional train-test division was employed, followed by model training and subsequent testing using the held-out test set. Upon examining the data in Table 5, it is evident that among the cross-validation configurations for *k* values of 2, 3, 4, and 5, the setting with *k* = 5 demonstrates the most effective performance, exhibiting the lowest WER at 23.62% and a CER of 4.12%. This indicates that the model benefits from increased exposure to training instances, which facilitates the acquisition of relevant linguistic patterns from the audio input. Conversely, when k=2 is used, the framework records the highest WER of 29.52% and a CER of 5.31%, indicating suboptimal learning. Furthermore, a comparison between the “Base” model and the 5-fold cross-validation method reveals that models achieve superior performance under the latter approach.

**Table 5:** Held-out test set performance for Wav2Vec2 XLSR-53 across different training configurations.

Training configuration	Test WER	Test CER	Eval loss
k = 2	29.52%	5.31%	0.3039
Base	25.67%	4.53%	0.2573
k = 3	25.98%	4.46%	0.2815
k = 4	25.11%	4.34%	0.2897
k = 5	23.62%	4.12%	0.2973

All models were evaluated on the same fixed 890-sentence held-out test set; *k* denotes the number of cross-validation folds used during training, not the evaluation set.

**Figure 2:**
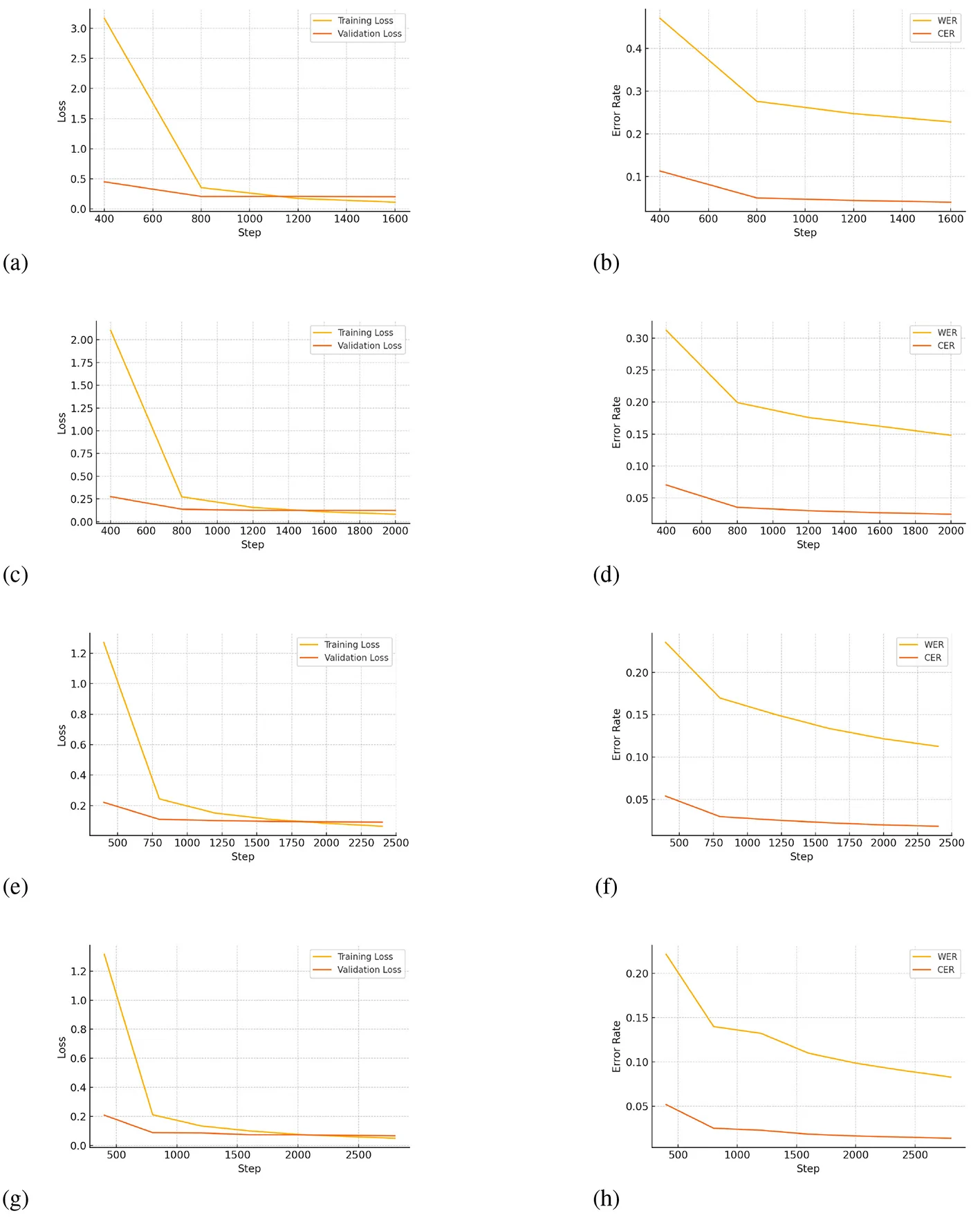
Wav2Vec2 XLSR-53: **(a, c, e, g)** Show training/validation loss curves, and **(b, d, f, h)** Show WER/CER curves for folds *k* = 2, 3, 4, 5. **(a)** Training and validation losses for fold *k* = 2. **(b)** WER and CER curves for fold *k* = 2. **(c)** Training and validation losses for fold *k* = 3. **(d)** WER and CER curves for fold *k* = 3. **(e)** Training and validation losses for fold *k* = 4. **(f)** WER and CER curves for fold *k* = 4. **(g)** Training and validation losses for fold *k* = 5. **(h)** WER and CER curves for fold *k* = 5.

Figures 2a,c, e, g illustrates the training and validation loss, and one can observe through the depicted graphs that both losses exhibit a downward progression. The observed decline in training and validation loss across each fold indicates that the model captures the underlying linguistic and temporal characteristics inherent in the Telugu speech data. In Figures 2b, d, f, h, the graphical representations of WER and CER illustrate that as training progresses, there is a notable reduction in both CER and WER relative to the number of training steps across the five-fold cross-validation. Particularly in the case of the fold *k* = 5 (refer to Figures 2g, h, there is a pronounced decline in training and validation losses as well as WER and CER values, indicating that the *k* = 5 configuration yields superior performance compared to the other folds.

The fold-wise summaries of the performance of the Wav2Vec2 XLSR-53 model for values of *k* = 2, 3, 4, and 5 are presented in Tables 6–9, respectively. In each table, the mean and standard deviation of training loss, validation loss, WER, and CER across the training steps are reported. In Table 6, for *k* = 2, fold-1 exhibits higher both training and validation losses, whereas fold-2 demonstrates lower loss values and error rates. For *k* = 3 (Table 7), the model depicts a consistent reduction in both WER and CER across folds, with fold-3 achieving the lowest error rates. In the case of *k* = 4 (Table 8), a decrease in both training and validation losses is observed from fold-1 to fold-4. Also, there is a decline in WER and CER, which indicates that additional folds contribute to more reliable performance. Finally, Table 9 presents the results for *k* = 5, where the model achieves the lowest losses and error rates in fold-5. Overall, these results illustrate that as *k* increases, the performance of Wav2Vec2 XLSR-53 becomes more consistent. To gain deeper insights into the model's performance across different training sets, the detailed results for each value of *k* = 2, 3, 4, and 5 are provided in Supplementary Tables 1–4, respectively.

**Table 6:** Fold-wise summary of Wav2Vec2 XLSR-53 performance (*k* = 2) on the k-fold validation folds.

Fold	Train loss	Val loss	WER	CER
Fold-1	1.7215 ± 2.5275	0.2181 ± 0.2123	0.4045 ± 0.2005	0.0582 ± 0.0632
Fold-2	0.1773 ± 0.0770	0.0589 ± 0.0043	0.1115 ± 0.0123	0.0160 ± 0.0016

**Table 7:** Fold-wise summary of Wav2Vec2 XLSR-53 performance (*k* = 3) on the k-fold validation folds.

Fold	Train loss	Val loss	WER	CER
Fold-1	1.6093 ± 2.4692	0.3027 ± 0.1693	0.4580 ± 0.1900	0.0722 ± 0.0562
Fold-2	0.1341 ± 0.0464	0.0495 ± 0.0036	0.0979 ± 0.0099	0.0139 ± 0.0017
Fold-3	0.0925 ± 0.0270	0.0202 ± 0.0027	0.0443 ± 0.0065	0.0061 ± 0.0010

**Table 8:** Fold-wise summary of Wav2Vec2 XLSR-53 performance (*k* = 4) on the k-fold validation folds.

Fold	Train loss	Val loss	WER	CER
Fold-1	0.9853 ± 1.8574	0.3100 ± 0.2004	0.4192 ± 0.1800	0.0659 ± 0.0543
Fold-2	0.1211 ± 0.0453	0.0463 ± 0.0064	0.0964 ± 0.0146	0.0149 ± 0.0021
Fold-3	0.0882 ± 0.0308	0.0228 ± 0.0056	0.0467 ± 0.0115	0.0067 ± 0.0019
Fold-4	0.0659 ± 0.0216	0.0114 ± 0.0041	0.0281 ± 0.0091	0.0034 ± 0.0013

**Table 9:** Fold-wise summary of Wav2Vec2 XLSR-53 performance (*k* = 5) on the k-fold validation folds.

Fold	Train loss	Val loss	WER	CER
Fold-1	1.5134 ± 2.0585	0.3216 ± 0.2642	0.4674 ± 0.2123	0.0862 ± 0.0728
Fold-2	0.1023 ± 0.0417	0.0446 ± 0.0074	0.0897 ± 0.0171	0.0136 ± 0.0025
Fold-3	0.0715 ± 0.0284	0.0209 ± 0.0072	0.0499 ± 0.0150	0.0060 ± 0.0024
Fold-4	0.0579 ± 0.0218	0.0116 ± 0.0045	0.0303 ± 0.0110	0.0040 ± 0.0016
Fold-5	0.0531 ± 0.0196	0.0083 ± 0.0034	0.0202 ± 0.0110	0.0025 ± 0.0011

### 4.3 Analysis on k-fold validation for Whisper-small

This portion delivers an in-depth analysis concerning the results obtained from building the Whisper-small framework by employing a 5-fold cross-validation methodology. It further provides the distinct effectiveness associated with individual folds corresponding to values *k* = 2, 3, 4, and 5, thereby providing an interpretative understanding of the operational characteristics of Whisper-small within the context of the specified Telugu language.

In Table 10, performance trends of the Whisper-small system are represented for CER, WER, and evaluation loss observed across different k-fold validation paradigms. The term “Base” in this setting denotes the utilization of a traditional train and test split, wherein training precedes evaluation against a retained test subset. The findings reveal that across the cross-validation settings for k-values of 2, 3, 4, and 5, the configuration with *k* = 5 manifests the most favorable outcome on the test dataset, yielding the minimum WER of 28.67% along with a CER of 5.48%. This trend implies enhanced learning efficiency when the model is exposed to a broader range of training examples, thereby improving its capacity to extract linguistic information from audio features. In contrast, at *k* = 2, the model exhibits inferior generalization, as evidenced by the WER of 32.05% and CER of 6.16%. Furthermore, when contrasting the traditional train–test split with the *k* = 5 cross-validation approach, the Whisper-small model exhibits comparable performance, with only marginal differences of 0.91% in WER and 0.33% in CER.

**Table 10:** Held-out test set performance for Whisper-small across different training configurations.

Model	WER	CER	Eval Loss
Base	27.76%	5.15%	0.1215
k = 2	32.05%	6.16%	0.1469
k = 3	29.72%	5.65%	0.1609
k = 4	29.40%	5.78%	0.1720
k = 5	28.67%	5.48%	0.1668

All models were evaluated on the same fixed 890-sentence held-out test set; *k* denotes the number of cross-validation folds used during training, not the evaluation set.

**Figure 3:**
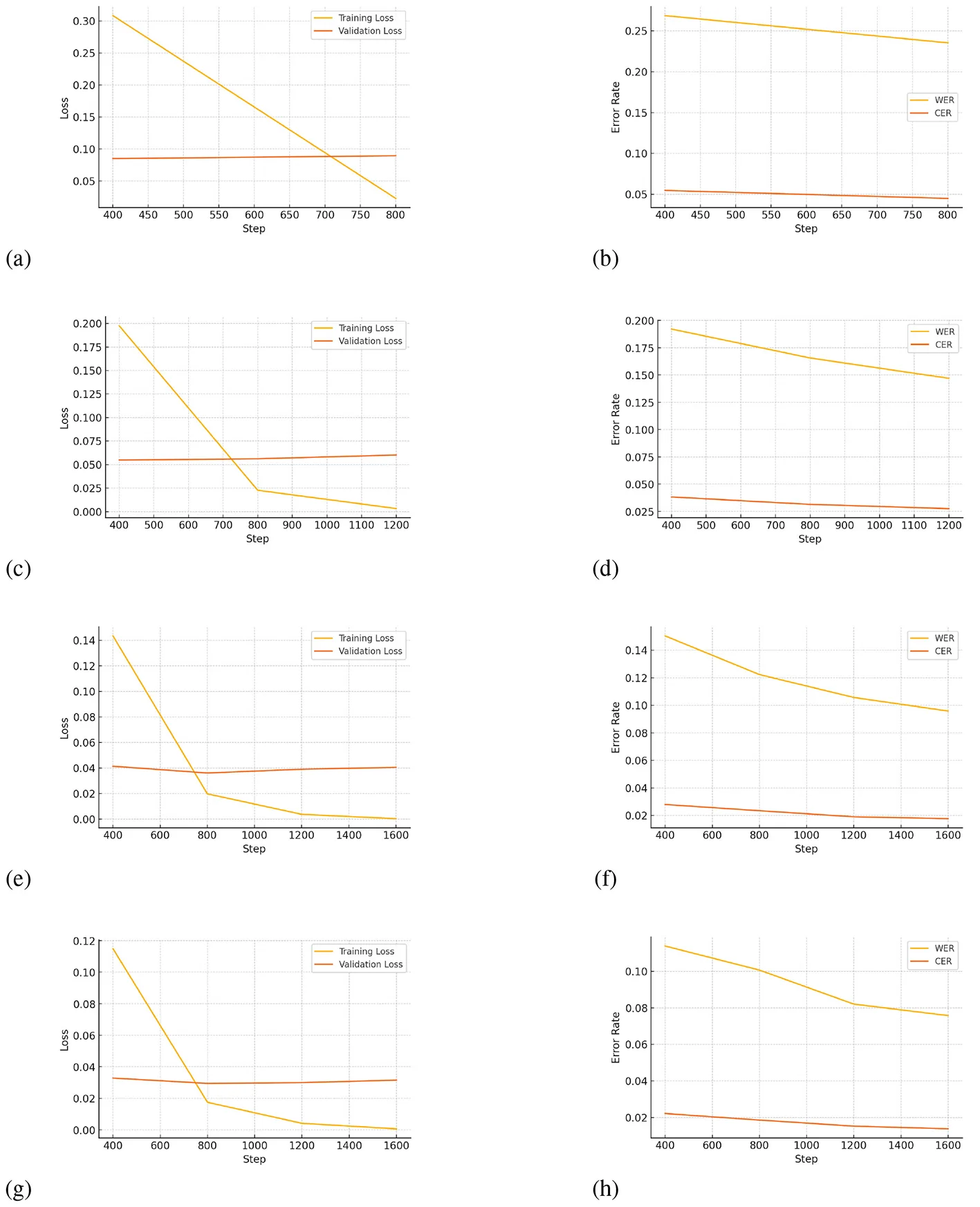
Whisper-small: **(a, c, e, g)** Show training/validation loss curves, and **(b, d, f, h)** Show WER/CER curves for folds *k* = 2, 3, 4, 5. **(a)** Training and validation losses for fold *k* = 2. **(b)** WER and CER curves for fold *k* = 2. **(c)** Training and validation losses for fold *k* = 3. **(d)** WER and CER curves for fold *k* = 3. **(e)** Training and validation losses for fold *k* = 4. **(f)** WER and CER curves for fold *k* = 4. **(g)** Training and validation losses for fold *k* = 5. **(h)** WER and CER curves for fold *k* = 5.

Figures 3a, c, e, g demonstrates that the training and validation losses demonstrate a consistent decline across every fold, suggesting that the model is adept at learning and encoding the temporal and linguistic structures embedded in Telugu speech data. Such a recurrent trend among the folds indicates minimal likelihood of overfitting. Moreover, the visual plots in Figures 3b, d, f, h associated with the CER and WER reveal that, with the progression of training steps, both CER and WER undergo a marked decrease. This pattern is clear in fold k = 5, where the graphs show a big drop in both loss and error, suggesting that the k = 5 setup works better than the other folds, thereby implying that the k = 5 setting outperforms other fold configurations.

Tables 11–14 present the fold-wise summary of the performance of the Whisper-small model across different values of *k* = 2, 3, 4, 5 in *k*. For *k* = 2 (Table 11), fold 1 exhibits higher WER and CER as compared to fold 2. For *k* = 3 (Table 12), fold 3 demonstrates low WER and CER. In case *k* = 4 (Table 13), there is a similar trend, where lower WER and CER values are observed in later folds. Finally, for *k* = 5 (Table 14), the results are the most consistent across folds with minimum values of WER and CER. The findings show that larger values of *k* yield more reliable performance in low-resource ASR settings. Comprehensive results for each value of *k* = 2, 3, 4, and 5 of Whisper-small model are presented in Supplementary Tables 5–8, respectively to provide a deeper understanding of the model's performance across different training steps of cross-validation.

**Table 11:** Fold-wise summary of Whisper-small performance (*k* = 2) on the k-fold validation folds.

Fold	Train loss	Val loss	WER	CER
Fold-1	0.2827 ± 0.2501	0.1593 ± 0.0066	0.4324 ± 0.0520	0.0877 ± 0.0147
Fold-2	0.0488 ± 0.0355	0.0155 ± 0.0001	0.0715 ± 0.0053	0.0119 ± 0.0006

**Table 12:** Fold-wise summary of Whisper-small performance (*k* = 3) on the k-fold validation folds.

Fold	Train loss	Val loss	WER	CER
Fold-1	0.1973 ± 0.2610	0.1482 ± 0.0096	0.3969 ± 0.0486	0.0727 ± 0.0142
Fold-2	0.0214 ± 0.0207	0.0195 ± 0.0029	0.0885 ± 0.0110	0.0141 ± 0.0018
Fold-3	0.0049 ± 0.0031	0.0036 ± 0.0012	0.0191 ± 0.0047	0.0030 ± 0.0009

**Table 13:** Fold-wise summary of Whisper-small performance (*k* = 4) on the k-fold validation folds.

Fold	Train loss	Val loss	WER	CER
Fold-1	0.1502 ± 0.2280	0.1408 ± 0.0111	0.3761 ± 0.0544	0.0747 ± 0.0126
Fold-2	0.0113 ± 0.0143	0.0079 ± 0.0016	0.0437 ± 0.0120	0.0078 ± 0.0023
Fold-3	0.0031 ± 0.0030	0.0067 ± 0.0041	0.0384 ± 0.0323	0.0047 ± 0.0035
Fold-4	0.0021 ± 0.0024	0.0035 ± 0.0019	0.0161 ± 0.0086	0.0026 ± 0.0016

**Table 14:** Fold-wise summary of Whisper-small performance (*k* = 5) on the k-fold validation folds.

Fold	Train loss	Val loss	WER	CER
Fold-1	0.1530 ± 0.2323	0.1368 ± 0.0106	0.3731 ± 0.0563	0.0750 ± 0.0132
Fold-2	0.0096 ± 0.0111	0.0072 ± 0.0021	0.0360 ± 0.0123	0.0058 ± 0.0019
Fold-3	0.0032 ± 0.0028	0.0043 ± 0.0026	0.0271 ± 0.0112	0.0044 ± 0.0017
Fold-4	0.0020 ± 0.0023	0.0040 ± 0.0022	0.0237 ± 0.0166	0.0031 ± 0.0020
Fold-5	0.0023 ± 0.0023	0.0030 ± 0.0011	0.0127 ± 0.0046	0.0020 ± 0.0005

Although the mean and standard deviation of the training data appear more favorable for Whisper-small compared to Wav2Vec2 XLSR-53. The reason is that the Whisper-small model's execution was halted at training step 1600 for *k* = 4 and *k* = 5, and at training steps 800 and 1200 for *k* = 2 and *k* = 3, respectively. Following these specific stopping points, the values for validation loss, training loss, WER, and CER showed an upward trend compared to earlier steps. This pattern is supported by the ablation study results presented in Table 4, which analyzes performance across different epochs. The performance of Whisper-small is not satisfactory on the openSLR testing set. Furthermore, when evaluated on unseen noisy data, Whisper-small demonstrates a further decline in performance relative to Wav2Vec2 XLSR-53 (refer to Table 15). These findings indicate that the Whisper-small model fails to converge and leads to overfitting.

**Table 15:** WER and CER with 95% CIs for Wav2Vec2 XLSR-53 and Whisper-small.

Model	WER (%)	CER (%)	WER CI (95%)	CER CI (95%)
Wav2Vec2 XLSR-53	23.62	4.12	22.1% – 25.4%	3.8% – 4.2%
Whisper-small	28.67	5.48	26.7% – 30.8%	4.9% – 5.7%

### 4.4 Comparison between Wav2Vec2 XLSR-53 and Whisper-small

**Figure 4:**
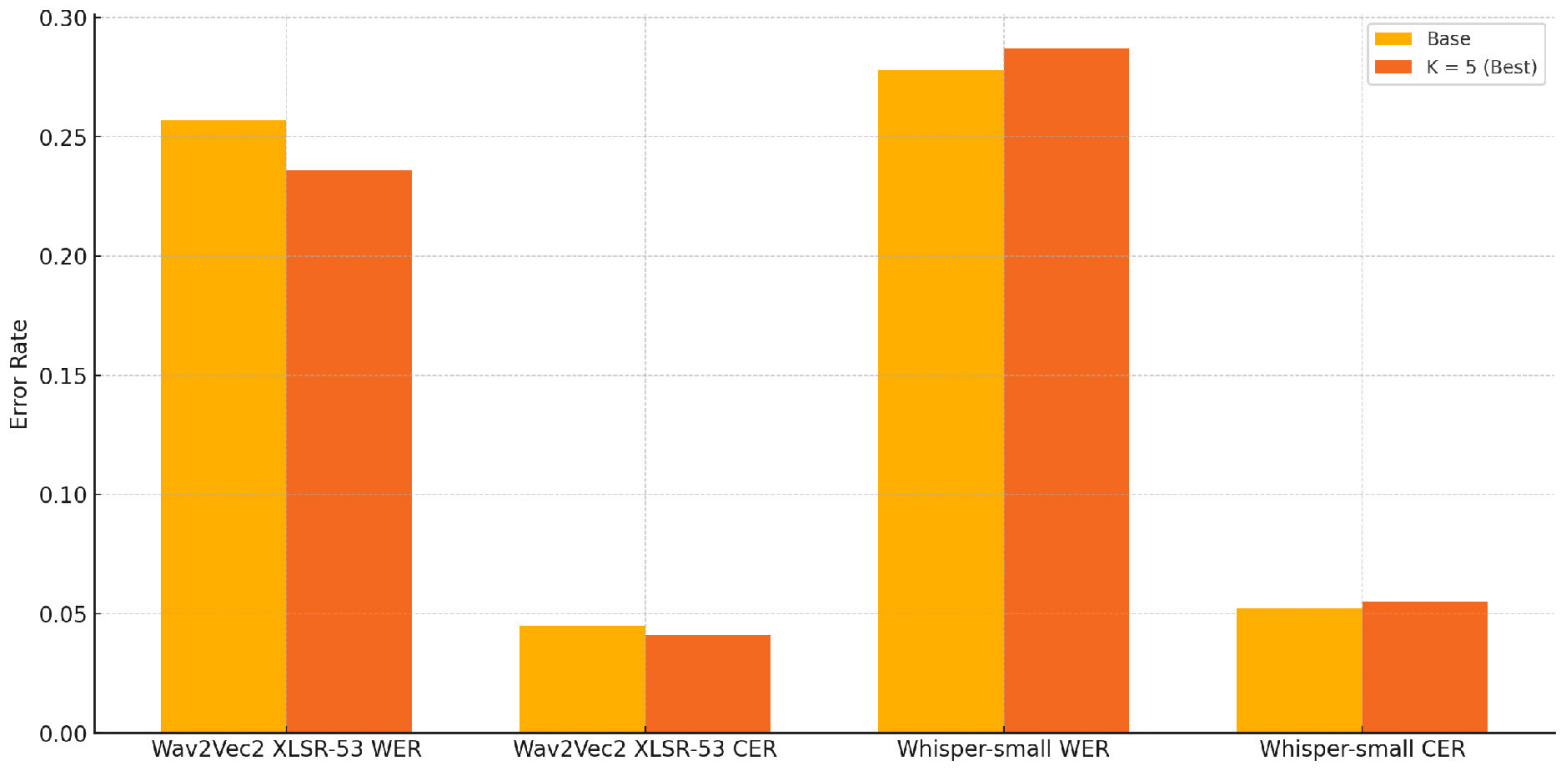
Comparison between the “Base” (train-test split, no cross-validation) strategy and the best-performing *k* = 5 fold cross-validation configuration for Wav2Vec2 XLSR-53 and Whisper-small models, evaluated on the fixed held-out test set.

Figure 4 illustrates a comparative analysis between the train-test split denoted herein as “Base” (conventional train-test split) and the 5-fold cross-validation across both Wav2Vec2 XLSR-53 and Whisper-small models. The graphical representation reveals that the 5-fold cross-validation technique surpasses the train-test split strategy in performance. For the Wav2Vec2 XLSR-53 framework, the train-test split approach yielded a WER of 25.67% and a CER of 4.53%, while applying 5-fold cross-validation achieved improved results, with a WER of 23.62% and a CER of 4.12%. In the context of the Whisper-small framework, the performance margins were comparatively similar between the two strategies. The train-test split method produced a WER of 27.76% and CER of 5.15%, whereas the 5-fold cross-validation approach recorded a slightly higher WER of 28.67% and CER of 5.48%.

Additionally, when evaluating the relative performance between the two models, the findings show the superiority of Wav2Vec2 XLSR-53 over Whisper-small. The models developed using the 5-fold cross-validation approach achieved a satisfactory WER and CER. The enhanced effectiveness of Wav2Vec2 XLSR-53 can be attributed to its training paradigm, which leverages SSL. This SSL technique enables the model to acquire rich phonetic features using minimal amounts of labeled data. On the other hand, the Whisper-small architecture is based on an encoder-decoder framework, which inherently introduces additional complexity and a stronger reliance on labeled datasets.

Wav2Vec2 XLSR-53 comprises approximately 316 million parameters, in contrast to Whisper-small, which contains 242 million parameters. Owing to its higher parameter count, Wav2Vec2 XLSR-53 demonstrates a greater capacity to capture the linguistic context and phonetic representations of the Telugu language. Also, Wav2Vec2 XLSR-53 is better suited for low-resource language scenarios when handling unlabeled data (Baevski et al., 2020). In our experiments, Wav2Vec2 XLSR-53 achieved a WER of 23.62% and a CER of 4.12%, whereas Whisper-small attained a WER of 28.67% and a CER of 5.48%. At both word and character levels, Wav2Vec2 XLSR-53 outperformed Whisper-small by margins of 5.05% and 1.36%, respectively, for the test dataset. The self-supervised learning (SSL) pretraining of Wav2Vec2 XLSR-53 enables effective learning of phonetic representations with minimal labeled data. In contrast, encoder-decoder architectures like Whisper-small rely more heavily on labeled data and introduce additional complexity. Wav2Vec2 XLSR-53's convolutional encoder, followed by a transformer-based context network dedicated to learning audio representations, reinforces its suitability for low-resource environments, as observed in our study (Conneau et al., 2020).

We observe that the 5-fold cross-validation approach yields a WER of 23.62% for Wav2Vec2 XLSR-53, compared to 25.67% obtained using the traditional train-test split. In contrast, for Whisper-small, the WER slightly increases to 28.67% under a 5-fold cross-validation approach, while 27.76% with the train-test split approach. This pattern likely arises from differences in pretraining and sensitivity to fine-tuning: Wav2Vec2 XLSR-53 (a self-supervised encoder) gains measurable benefit from training across multiple folds and thus more exposure to varied labeled data during fine-tuning, while Whisper-small, trained on a massive unlabeled corpus and is more sensitive to fold composition, hyperparameter stability, and evaluation settings. In addition, k-fold averages can be influenced by a small number of “hard” folds (speaker, accent, or noise concentration), random seed effects, and early-stopping differences.

### 4.5 Model evaluation observations

In this section, we present the evaluation of two distinct models: Wav2Vec2 XLSR-53 and Whisper-small. The analysis incorporates statistical techniques, including confidence interval (CI) estimation. Furthermore, the evaluation considers both noisy data and an OpenSLR held-out test set. Detailed descriptions of these aspects are provided in the subsequent subsections.

#### 4.5.1 Model statistical evaluation

The fine-tuned models were evaluated using 95% confidence intervals (CIs) for both WER and CER, computed via bootstrap resampling on the held-out test set, thereby providing a more rigorous statistical assessment of model performance, as presented in Table 15. These CIs indicate the possible range of the models' true error rates and add credibility to the observed improvements. The results show that for Wav2Vec2 XLSR-53, the CI are relatively narrow, indicating consistent performance across samples. In contrast, Whisper-small shows slightly wider intervals, reflecting greater variability in performance. The results are summarized below:

#### 4.5.2 Model evaluation with OpenSLR testing set and unseen noisy data

Table 16 presents a comparative analysis of the performance of Wav2Vec2 XLSR-53 and Whisper-small on two evaluation datasets: the OpenSLR held-out test set and an unseen noisy dataset constructed by mixing clean Telugu test utterances with environmental sound clips from the ESC-50 dataset (Piczak, 2015). This noisy evaluation was designed as a robustness check to assess whether the fine-tuned models retain reasonable transcription quality under background acoustic disturbance. On the held-out test set, Wav2Vec2 XLSR-53 reports satisfactory performance, with a WER of 23.62% and a CER of 4.12%, while Whisper-small records a WER of 28.67% and a CER of 5.48%. On the unseen noisy dataset, both models demonstrate a decline in performance relative to the clean held-out test set, consistent with the expected effect of background noise on ASR accuracy. Wav2Vec2 XLSR-53 achieves a WER of 30.14% and a CER of 5.80% under noisy conditions, while Whisper-small achieves a WER of 37.95% and a CER of 8.47%. These results indicate that both fine-tuned models retain a degree of transcription quality when evaluated on this preliminary noisy set, with Wav2Vec2 XLSR-53 showing comparatively smaller degradation than Whisper-small.

**Table 16:** WER and CER for Wav2Vec2 XLSR-53 and Whisper-small on the OpenSLR held-out test set and an unseen noisy dataset.

Dataset	Model	WER (%)	CER (%)
OpenSLR testing set	Wav2Vec2 XLSR-53	23.62	4.12
	Whisper-small	28.67	5.48
Unseen noisy set	Wav2Vec2 XLSR-53	30.14	5.80
	Whisper-small	37.95	8.47

**Figure 5:**
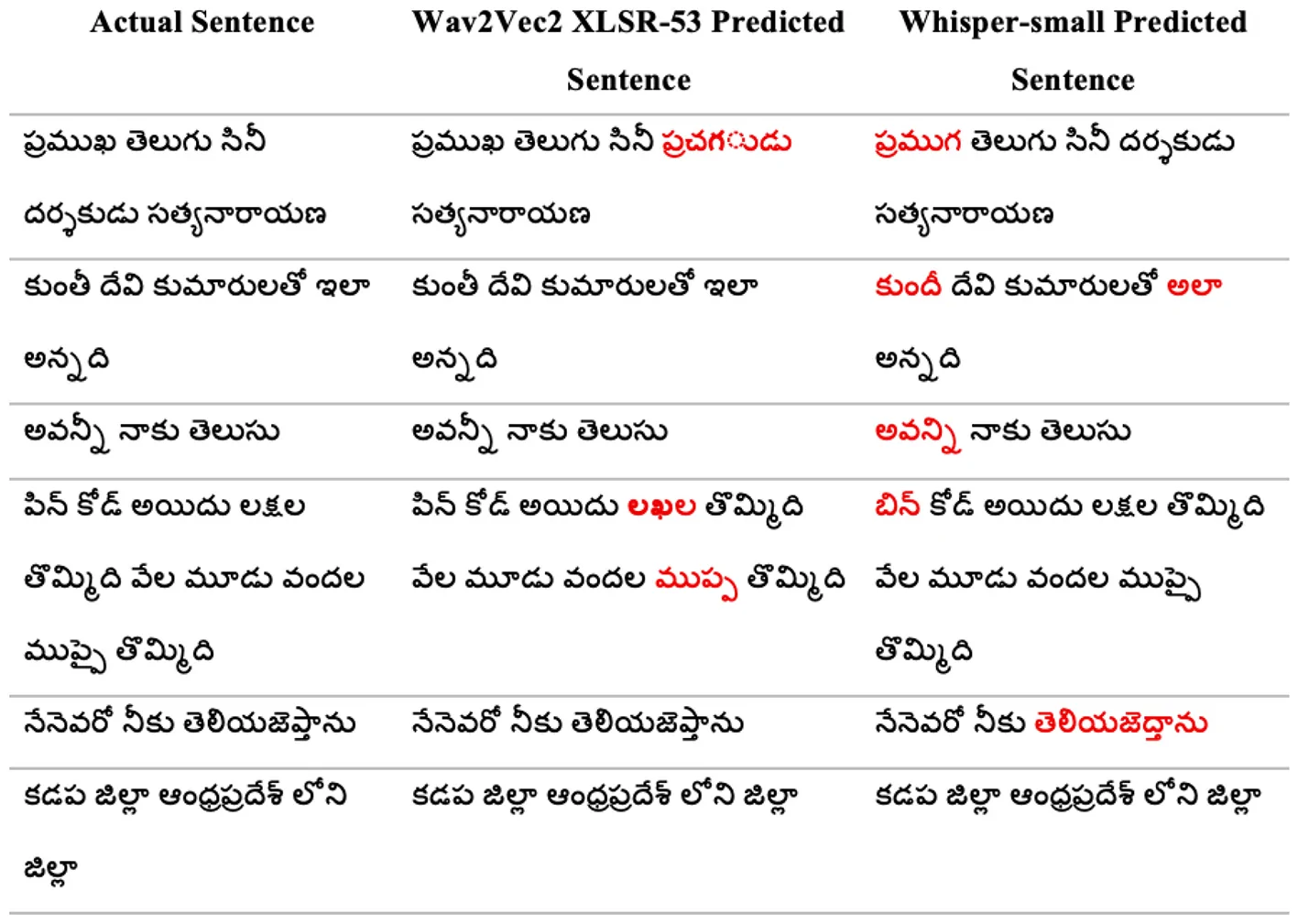
Actual sentence vs. predicted sentence using Wav2Vec2 XLSR-53 and Whisper-small for the OpenSLR held-out test set (red-colored words indicate that the model mispredicts).

**Figure 6:**
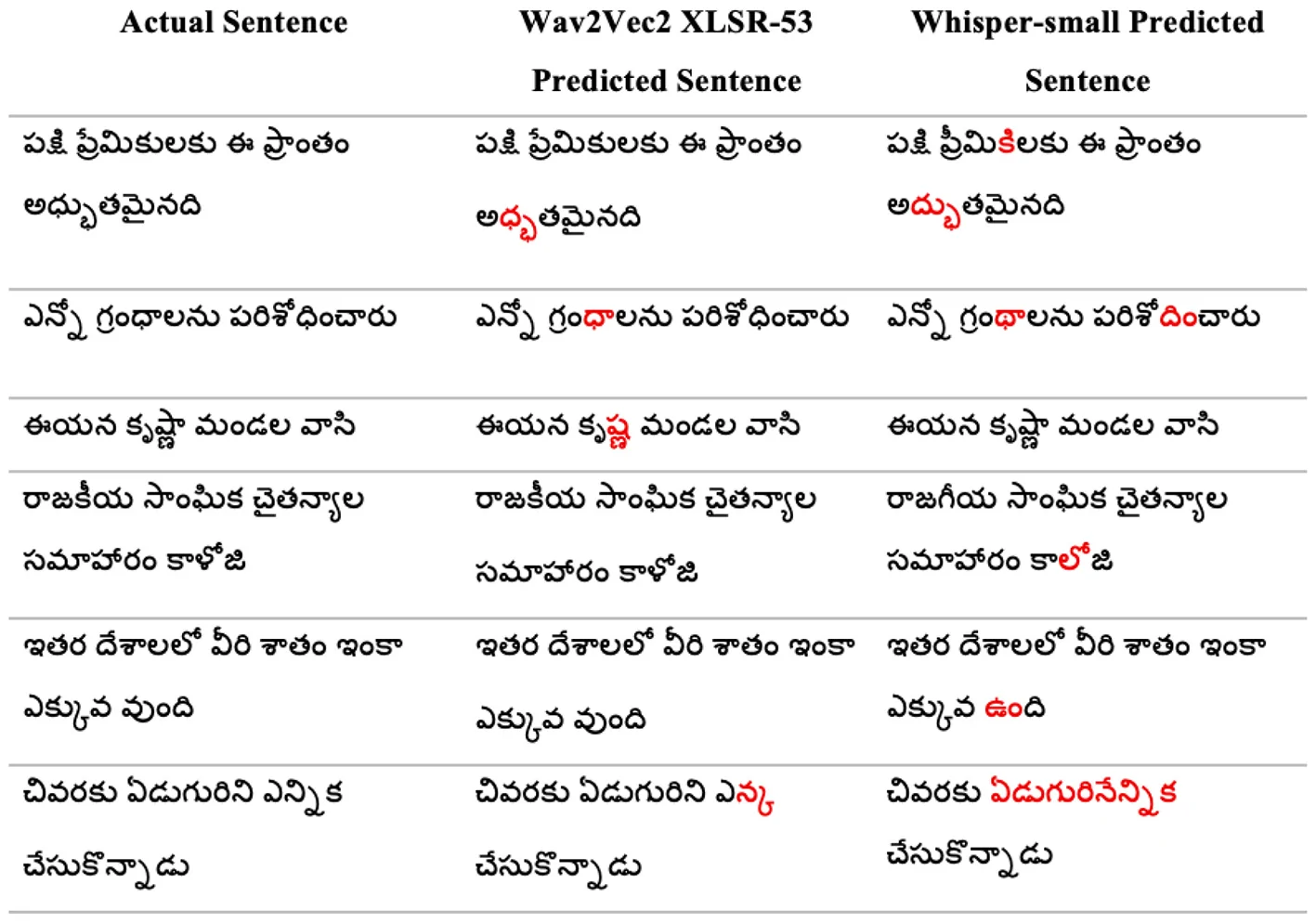
Actual sentence vs. predicted sentence using Wav2Vec2 XLSR-53 and Whisper-small for unseen noisy data (red-colored words indicate that the model mispredicts).

Both Wav2Vec2 XLSR-53 and Whisper-small were evaluated on six randomly selected clips from the OpenSLR held-out test set (refer to Figure 5). Wav2Vec2 XLSR-53 produced highly intelligible transcriptions, often aligning with the actual sentence in simpler segments, though errors occurred in complex or compound expressions where words were occasionally omitted or rearranged. Importantly, repeated sequences were transcribed consistently, indicating effective pattern learning, and these mismatches could be reduced with further tuning or a larger Telugu corpus. Similarly, Whisper-small performed well on complete phrases and continuous speech, reflecting strong language modeling, with its main errors involving the splitting of compound words and substitutions that subtly altered meaning. Overall, both models generated transcripts that closely resembled the actual text, suggesting potential for further improvements through domain-specific training. Also, the models were evaluated on unseen noisy data (refer to Figure 6). The models preserve the overall sentence structure and meaning, demonstrating their ability to handle challenging acoustic environments. Despite background disturbances, the transcriptions remain comprehensible, with only minor variations in word endings or phonetically similar terms. This highlights the reliability of both models for practical applications where noise is unavoidable.

#### 4.5.3 Qualitative analysis of recognition errors

An in-depth inspection of the misrecognition illustrated in Figures 5, 6 reveals three predominant error categories:

1. The Telugu language often combines several lexical units into a unified compound structure. The examined models occasionally encountered limitations in accurately decoding such constructions, resulting in the merging of word boundaries.
2. Regional pronunciation shifts led to frequent misrecognition of speech sounds. This phenomenon was pronounced in polysyllabic lexical items.
3. Linguistic elements reliant on contextual cues, such as idiomatic phrases, were at times inaccurately omitted. This highlights the inherent difficulty of encoding sociolinguistic knowledge within STT systems.

### 4.6 Comparison to existing studies

Table 17 presents a comparative analysis between the proposed model and existing Telugu ASR studies. Among studies evaluated on the OpenSLR Telugu corpus, the proposed models—Wav2Vec2 XLSR-53, which achieves a WER of 23.62% and a CER of 4.12%, and Whisper-small, which attains a WER of 28.67% and a CER of 5.48%—both outperform the corresponding fine-tuned models reported by Jain and Bhowmick (2025) on the same dataset. Relative to studies conducted on different corpora, Krishna (2021) reports a lower WER (27.83%) using a Conformer-based, grapheme-level system trained on a distinct 40-h dataset, while Diwan et al. (2021) and Chowdary et al. (2024) report higher WER on their respective custom and combined datasets. These results indicate that the proposed fine-tuning approach is competitive within the OpenSLR-based comparison group, while cross-dataset comparisons should be interpreted with the caveat noted above.

**Table 17:** Comparison of proposed fine-tuned Wav2Vec2 XLSR-53 and Whisper-small models with existing Telugu ASR studies.

References	Dataset	Duration	Method	WER (%)	CER (%)
Diwan et al. (2021)	MUCS 2021 (interspeech challenge)	Telugu subset of ~600 h multilingual corpus (7 languages)	Hybrid DNN-HMM / end-to-end (challenge baseline)	28.71 (on Telugu language)	NR
Krishna (2021)	Microsoft/speechOcean.com (INTERSPEECH 2018 challenge)	40 h	Conformer + dual-decoder (grapheme + phoneme, multi-task)	25.28	6.18
Chowdary et al. (2024)	OpenSLR + FLEURS	5.71 h (OpenSLR) + 3085 utt. (FLEURS)	Whisper	61.20	NR
Jain and Bhowmick (2025)	OpenSLR	5.69 h	Fine-tuned Wav2Vec2 XLSR-53	28.67	4.92
Jain and Bhowmick (2025)	OpenSLR	5.69 h	Fine-tuned Whisper-Small	30.04	10.31
**Proposed work**	**OpenSLR**	**6 h**	**Fine-tuned Wav2Vec2 XLSR-53**	**23.62**	**4.12**
**Proposed work**	**OpenSLR**	**6 h**	**Fine-tuned Whisper-Small**	**28.67**	**5.48**

WER/CER values are not directly comparable across studies using different datasets. Bold values indicate the best performance.

## 5 Conclusion

This study endeavored to enhance STT frameworks tailored to the low-resource language, Telugu. A comparative evaluation involving the architectures of Wav2Vec2 XLSR-53 and Whisper-small was fine-tuned for the Telugu STT task. In addition, a 5-fold cross-validation methodology was conducted for both models to evaluate the effectiveness of fine-tuned Wav2Vec2 XLSR-53 and Whisper-small architectures. The findings indicated that the 5-fold cross-validation strategy yielded effective results for Wav2Vec2 XLSR-53, whereas Whisper-small produced CER and WER to the conventional train-test split. Furthermore, we evaluated the model on unseen noisy data, and the results were found to be satisfactory. Additionally, we achieved a 95% confidence interval for both the WER and CER. The dataset has limitations in that it includes speakers only from a restricted set of Telugu-speaking regions. Additionally, speaker overlap across folds prevents the models from capturing the full range of dialectal variation within the Telugu language. In the future, exploring the impact of various augmentation techniques applied to audio samples could facilitate insights into their influence on model accuracy. Also, a complete SNR-based noise robustness analysis, evaluating model performance across a systematic range of SNR levels (e.g., 0 dB, 5 dB, 10 dB, 15 dB) and multiple ESC-50 noise categories to precisely characterize how varying noise intensities impact the fine-tuned models' performance, can be explored. Additionally, extending this analysis to multiple low-resource Indian languages may support the development of more generalized STT frameworks. The implementation details and resources of this work are accessible via the GitHub repository: Code: SST-Telugu-K-Fold-Wav2Vec-Whisper.

## Data Availability

The original contributions presented in the study are included in the article/Supplementary material, further inquiries can be directed to the corresponding author.

## References

[B1] Abdullah A. A. Karim S. H. T. Ahmed S. A. Tariq K. R. Rashid T. A. (2025). Speaker diarization for low resource languages through wav2vec fine tuning. arXiv preprint, abs/2504.18582. doi: 10.48550/arXiv.2504.18582

[B2] Abdullah A. A. Tabibian S. Veisi H. Mahmudi A. Rashid T. A. (2024). End to end transformer based automatic speech recognition for northern kurdish: a pioneering approach. arXiv preprint, abs/2410.16330. doi: 10.48550/arXiv.2410.16330

[B3] Abdullah A. A. Veisi H. (2022). Central kurdish automatic speech recognition using deep learning. J. Univ. Anbar Pure Sci. 16, 108–118. doi: 10.37652/juaps.2022.176500

[B4] Ahmad Dar M. Pushparaj J. (2025). Machine learning and deep learning approaches for accent recognition: a review. IEEE Access (Piscataway, NJ: Institute of Electrical and Electronics Engineers (IEEE)) 13, 51527–51550. doi: 10.1109/ACCESS.2025.3552935

[B5] Alsayadi H. A. Abdelhamid A. A. Hegazy I. Fayed Z. T. (2021). Arabic speech recognition using end-to-end deep learning. IET Signal Process. 15, 521–534. doi: 10.1049/sil2.12057

[B6] Ardila R. Branson M. Davis K. Kohler M. Meyer J. Henretty M. . (2020). “Common voice: a massively-multilingual speech corpus,” in Proceedings of the twelfth language resources and evaluation conference (Marseille: European Language Resources Association), 4218–4222.

[B7] Baevski A. Zhou H. Mohamed A. Auli M. (2020). wav2vec 2.0: a framework for self-supervised learning of speech representations. doi: 10.48550/ARXIV.2006.11477

[B8] Chadha H. S. Shah P. Dhuriya A. Chhimwal N. Gupta A. Raghavan V. (2022). Code switched and code mixed speech recognition for indic languages. doi: 10.48550/ARXIV.2203.16578

[B9] Chowdary D. Ganesan R. Dabbara H. Jyothish Lal G. Premjith B. (2024). Transformer-Based Multilingual Automatic Speech Recognition (ASR) Model for Dravidian Languages, chap. 13. Chichester: John Wiley & Sons, Ltd., 259–273. doi: 10.1002/9781394214624.ch13

[B10] Conneau A. Baevski A. Collobert R. Mohamed A. Auli M. (2020). Unsupervised cross-lingual representation learning for speech recognition. doi: 10.48550/ARXIV.2006.13979

[B11] Dar M. A. Pushparaj J. (2024). “Hybrid architecture cnn-blstm for automatic speech recognition,” in 2024 3rd International conference on artificial intelligence for internet of things (AIIoT), 1–4. doi: 10.1109/AIIoT58432.2024.10574740

[B12] Dar M. A. Pushparaj J. (2025). Bi-directional lstm-based isolated spoken word recognition for kashmiri language utilizing mel-spectrogram feature. Appl. Acoust. 231:110505. doi: 10.1016/j.apacoust.2024.110505

[B13] Diwan A. Vaideeswaran R. Shah S. Singh A. Raghavan S. Khare S. . (2021). “Mucs 2021: multilingual and code-switching asr challenges for low resource indian languages,” in Proceedings of interspeech 2021 (Brisbane, QLD: ISCA), 2446–2450. doi: 10.21437/Interspeech.2021-1339

[B14] Fathima N. Patel T. C M. Iyengar A. (2018). “Tdnn-based multilingual speech recognition system for low resource indian languages,” in Proceedings of interspeech 2018 (Hyderabad: ISCA), 3197–3201. doi: 10.21437/Interspeech.2018-2117

[B15] Ghadekar P. Jhanwar K. Sivanandan A. Shetty T. Karpe A. Khushalani P. (2023). “Asr for indian regional language using nvidia's nemo toolkit,” in WOMEN IN PHYSICS: 7th IUPAP international conference on women in physics, vol. 3040 (Melbourne, VIC: AIP Publishing), 020004. doi: 10.1063/5.0178629

[B16] Graves A. Fernández S. Gomez F. Schmidhuber J. (2006). “Connectionist temporal classification: labelling unsegmented sequence data with recurrent neural networks,” in Proceedings of the 23rd international conference on machine learning (ICML '06) (Pittsburgh, PA: ACM Press), 369–376. doi: 10.1145/1143844.1143891

[B17] He F. Chu S.-H. C. Kjartansson O. Rivera C. Katanova A. Gutkin A. . (2020). “Open-source multi-speaker speech corpora for building Gujarati, Kannada, Malayalam, Marathi, Tamil and Telugu speech synthesis systems,” in Proceedings of the twelfth language resources and evaluation conference (Marseille: European Language Resources Association (ELRA)), 6494–6503.

[B18] Islam J. Mubassira M. Islam M. R. Das A. K. (2019). “A speech recognition system for bengali language using recurrent neural network,” in 2019 IEEE 4th international conference on computer and communication systems (ICCCS) (Singapore: IEEE), 73–76. doi: 10.1109/CCOMS.2019.8821629

[B19] Jain P. Bhowmick A. (2025). Comparative performance analysis of end-to-end asr models on Indo-Aryan and Dravidian languages within India's linguistic landscape. EURASIP J. Audio, Speech Music Process. 2025:10. doi: 10.1186/s13636-025-00395-5

[B20] Karan B. Sahoo J. Sahu P. K. (2015). “Automatic speech recognition based odia system,” in 2015 International conference on microwave, optical and communication engineering (ICMOCE) (Bhubaneswar: IEEE), 353–356. doi: 10.1109/ICMOCE.2015.7489765

[B21] Kaur A. Singh A. (2016). “Power-normalized cepstral coefficients (pncc) for punjabi automatic speech recognition using phone-based modelling in htk,” in Proceedings of the 2016 2nd international conference on applied and theoretical computing and communication technology (iCATccT) (Bangalore: IEEE), 372–375. doi: 10.1109/ICATCCT.2016.7912026

[B22] Krishna D. N. (2021). Multilingual speech recognition for low-resource indian languages using multi-task conformer. doi: 10.48550/ARXIV.2109.03969

[B23] Kumar A. Aggarwal R. K. (2020). Hindi speech recognition using time delay neural network acoustic modeling with i-vector adaptation. Int. J. Speech Technol. 25, 67–78. doi: 10.1007/s10772-020-09757-0

[B24] Kumar A. Mittal V. (2021). Hindi speech recognition in noisy environment using hybrid technique. Int. J. Inform. Technol. 13, 483–492. doi: 10.1007/s41870-020-00586-7

[B25] Kumari A. Verma S. A. Singh E. Toppo A. C. Paul S. Bhattacharjee V. (2024). “An end-to-end deep learning based continuous speech recognition in Kannada,” in Proceedings of the TIM22 physics conference, vol. 3181 (Hyderabad: AIP Publishing), 050001. doi: 10.1063/5.0214118

[B26] Majhi M. K. Saha S. K. (2024). An automatic speech recognition system in odia language using attention mechanism and data augmentation. Int. J. Speech Technol. 27, 717–728. doi: 10.1007/s10772-024-10132-6

[B27] O'Shaughnessy D. (2008). Invited paper: automatic speech recognition: history, methods and challenges. Pattern Recogn. 41, 2965–2979. doi: 10.1016/j.patcog.2008.05.008

[B28] Piczak K. J. (2015). “ESC: dataset for environmental sound classification,” in Proceedings of the 23rd annual ACM conference on multimedia (Brisbane: ACM Press), 1015–1018. doi: 10.1145/2733373.2806390

[B29] Pinto D. Arnau J.-M. González A. (2022). Asrpu: a programmable accelerator for low-power automatic speech recognition. doi: 10.48550/ARXIV.2202.04971

[B30] Pratap V. Xu Q. Sriram A. Synnaeve G. Collobert R. (2020). Mls: a large-scale multilingual dataset for speech research. arXiv, abs/2012.03411. doi: 10.48550/ARXIV.2012.03411

[B31] Radford A. Kim J. W. Xu T. Brockman G. McLeavey C. Sutskever I. (2022). Robust speech recognition via large-scale weak supervision. doi: 10.48550/ARXIV.2212.04356

[B32] Reddy S. S. Mnoj S. K. A. Rao K. P. (2025). DNNT (deep neural network for Telugu): a framework for speech recognition of Telugu language with parallel computing approach. Int. J. Speech Technol. 28, 341–349. doi: 10.1007/s10772-025-10186-0

[B33] Salesky E. Wiesner M. Bremerman J. Cattoni R. Negri M. Turchi M. . (2021). The multilingual tedx corpus for speech recognition and translation. doi: 10.48550/ARXIV.2102.01757

[B34] Saraswathi S. Geetha T. (2004). “Implementation of Tamil speech recognition system using neural networks,” in Advances in neural networks - *ISNN 2004* (Berlin; Heidelberg: Springer), 169–176. doi: 10.1007/978-3-540-30176-9_22

[B35] Shi X. Yu F. Lu Y. Liang Y. Feng Q. Wang D. . (2021). “The accented english speech recognition challenge 2020: open datasets, tracks, baselines, results and methods,” in ICASSP 2021 - *2021 IEEE international conference on acoustics, speech and signal processing (ICASSP)* (Toronto, ON: IEEE), 6878–6882. doi: 10.1109/ICASSP39728.2021.9413386

[B36] Song Z. (2019). English speech recognition based on deep learning with multiple features. Computing 102, 663–682. doi: 10.1007/s00607-019-00753-0

[B37] Yu F.-H. Chen K.-Y. Lu K.-H. (2022). Non-autoregressive asr modeling using pre-trained language models for chinese speech recognition. IEEE/ACM Trans. Audio Speech Lang. Process. 30, 1474–1482. doi: 10.1109/TASLP.2022.3166400

