# Supplementary material for: Exploring Wav2Vec2 XLSR-53 and Whisper-small for Telugu language speech-to-text: a fine-tuning approach
Source: Front Artif Intell. 2026 Sep 9;9:1883049. doi: 10.3389/frai.2026.1883049 (PMC13597755; doi:10.3389/frai.2026.1883049)
Supplement: Supplementary file 1 [file Table_1.docx]

# SUPPLEMENTARY MATERIALS

**Table 1.** Performance analysis of Wav2Vec2 XLSR-53 for fold k = 2 on the k-fold validation folds.

| **Fold 1** | | | | |
| --- | --- | --- | --- | --- |
| Step | Train Loss | Val Loss | WER | CER |
| 400 | 6.0426 | 0.8401 | 0.8321 | 0.2113 |
| 800 | 0.5036 | 0.3432 | 0.4328 | 0.0824 |
| 1200 | 0.2137 | 0.3474 | 0.3881 | 0.0723 |
| **1600** | **0.1267** | **0.3417** | **0.3652** | **0.0666** |
| **Fold 2** | | | | |
| Step | Train Loss | Val Loss | WER | CER |
| 400 | 0.2954 | 0.0543 | 0.1086 | 0.0151 |
| 800 | 0.1973 | 0.0632 | 0.1196 | 0.0181 |
| 1200 | 0.1273 | 0.0626 | 0.1066 | 0.0165 |
| **1600** | **0.0904** | **0.0557** | **0.0911** | **0.0140** |
