# Supplementary material for: Exploring Wav2Vec2 XLSR-53 and Whisper-small for Telugu language speech-to-text: a fine-tuning approach
Source: Front Artif Intell. 2026 Sep 9;9:1883049. doi: 10.3389/frai.2026.1883049 (PMC13597755; doi:10.3389/frai.2026.1883049)
Supplement: Supplementary file 2 [file Table_2.docx]

# SUPPLEMENTARY MATERIALS

**Table 2.** Performance analysis of Wav2Vec2 XLSR-53 for fold k = 3 on the k-fold validation folds.

| **Fold 1** | | | | |
| --- | --- | --- | --- | --- |
| Step | Train Loss | Val Loss | WER | CER |
| 400 | 6.0161 | 0.7597 | 0.7857 | 0.1910 |
| 800 | 0.5127 | 0.3379 | 0.4373 | 0.0835 |
| 1200 | 0.2477 | 0.2988 | 0.3809 | 0.0693 |
| 1600 | 0.1544 | 0.3051 | 0.3554 | 0.0630 |
| **2000** | **0.1151** | **0.3119** | **0.3258** | **0.0582** |
| **Fold 2** | | | | |
| Step | Train Loss | Val Loss | WER | CER |
| 400 | 0.1957 | 0.0484 | 0.1068 | 0.0148 |
| 800 | 0.1790 | 0.0522 | 0.1060 | 0.0154 |
| 1200 | 0.1271 | 0.0516 | 0.0984 | 0.0148 |
| 1600 | 0.0970 | 0.0499 | 0.0922 | 0.0130 |
| **2000** | **0.0719** | **0.0432** | **0.0825** | **0.0113** |
| **Fold 3** | | | | |
| Step | Train Loss | Val Loss | WER | CER |
| 400 | 0.0992 | 0.0198 | 0.0437 | 0.0057 |
| 800 | 0.1295 | 0.0226 | 0.0544 | 0.0075 |
| 1200 | 0.0962 | 0.0240 | 0.0482 | 0.0069 |
| 1600 | 0.0754 | 0.0174 | 0.0392 | 0.0053 |
| **2000** | **0.0622** | **0.0170** | **0.0359** | **0.0049** |
