# Supplementary material for: Exploring Wav2Vec2 XLSR-53 and Whisper-small for Telugu language speech-to-text: a fine-tuning approach
Source: Front Artif Intell. 2026 Sep 9;9:1883049. doi: 10.3389/frai.2026.1883049 (PMC13597755; doi:10.3389/frai.2026.1883049)
Supplement: Supplementary file 3 [file Table_3.docx]

# SUPPLEMENTARY MATERIALS

**Table 3.** Performance analysis of Wav2Vec2 XLSR-53 for fold k = 4 on the k-fold validation folds.

| **Fold 1** | | | | |
| --- | --- | --- | --- | --- |
| Step | Train Loss | Val Loss | WER | CER |
| 400 | 4.7476 | 0.8116 | 0.7828 | 0.1935 |
| 800 | 0.5593 | 0.3368 | 0.4637 | 0.0881 |
| 1200 | 0.2814 | 0.3172 | 0.4092 | 0.0747 |
| 1600 | 0.1797 | 0.2986 | 0.3594 | 0.0640 |
| 2000 | 0.1341 | 0.3000 | 0.3356 | 0.0582 |
| **2400** | **0.1000** | **0.2974** | **0.3185** | **0.0549** |
| **Fold 2** | | | | |
| Step | Train Loss | Val Loss | WER | CER |
| 400 | 0.1738 | 0.0373 | 0.0774 | 0.0110 |
| 800 | 0.1765 | 0.0532 | 0.1173 | 0.0167 |
| 1200 | 0.1296 | 0.0522 | 0.1056 | 0.0159 |
| 1600 | 0.1055 | 0.0511 | 0.1005 | 0.0153 |
| 2000 | 0.0831 | 0.0467 | 0.0906 | 0.0133 |
| **2400** | **0.0641** | **0.0443** | **0.0881** | **0.0125** |
| **Fold 3** | | | | |
| Step | Train Loss | Val Loss | WER | CER |
| 400 | 0.0913 | 0.0269 | 0.0565 | 0.0080 |
| 800 | 0.1341 | 0.0305 | 0.0587 | 0.0088 |
| 1200 | 0.1050 | 0.0216 | 0.0516 | 0.0073 |
| 1600 | 0.0828 | 0.0238 | 0.0470 | 0.0069 |
| 2000 | 0.0644 | 0.0196 | 0.0389 | 0.0053 |
| **2400** | **0.0514** | **0.0162** | **0.0283** | **0.0040** |
| **Fold 4** | | | | |
| Step | Train Loss | Val Loss | WER | CER |
| 400 | 0.0727 | 0.0106 | 0.0247 | 0.0033 |
| 800 | 0.1042 | 0.0167 | 0.0388 | 0.0054 |
| 1200 | 0.0875 | 0.0145 | 0.0357 | 0.0048 |
| 1600 | 0.0682 | 0.0107 | 0.0281 | 0.0038 |
| 2000 | 0.0520 | 0.0083 | 0.0212 | 0.0030 |
| **2400** | **0.0425** | **0.0068** | **0.0158** | **0.0023** |
