# Supplementary material for: Exploring Wav2Vec2 XLSR-53 and Whisper-small for Telugu language speech-to-text: a fine-tuning approach
Source: Front Artif Intell. 2026 Sep 9;9:1883049. doi: 10.3389/frai.2026.1883049 (PMC13597755; doi:10.3389/frai.2026.1883049)
Supplement: Supplementary file 4 [file Table_4.docx]

# SUPPLEMENTARY MATERIALS

**Table 4.** Performance analysis of Wav2Vec2 XLSR-53 for fold k = 5 on the k-fold validation folds.

| **Fold 1** | | | | |
| --- | --- | --- | --- | --- |
| Step | Train Loss | Val Loss | WER | CER |
| 400 | 6.2238 | 0.9596 | 0.9009 | 0.2314 |
| 800 | 0.5731 | 0.3235 | 0.4464 | 0.0879 |
| 1200 | 0.2884 | 0.3143 | 0.4179 | 0.0771 |
| 1600 | 0.1885 | 0.2723 | 0.3455 | 0.0621 |
| 2000 | 0.1342 | 0.2840 | 0.3202 | 0.0561 |
| 2400 | 0.1073 | 0.2811 | 0.2999 | 0.0529 |
| **2800** | **0.0861** | **0.2711** | **0.2900** | **0.0499** |
| **Fold 2** | | | | |
| Step | Train Loss | Val Loss | WER | CER |
| 400 | 0.1505 | 0.0371 | 0.0884 | 0.0120 |
| 800 | 0.1682 | 0.0564 | 0.1143 | 0.0178 |
| 1200 | 0.1281 | 0.0542 | 0.1029 | 0.0160 |
| 1600 | 0.1046 | 0.0519 | 0.1007 | 0.0145 |
| 2000 | 0.0833 | 0.0438 | 0.0856 | 0.0129 |
| 2400 | 0.0695 | 0.0394 | 0.0798 | 0.0114 |
| **2800** | **0.0555** | **0.0348** | **0.0686** | **0.0104** |
| **Fold 3** | | | | |
| Step | Train Loss | Val Loss | WER | CER |
| 400 | 0.0847 | 0.0213 | 0.0590 | 0.0072 |
| 800 | 0.1263 | 0.0249 | 0.0539 | 0.0076 |
| 1200 | 0.0969 | 0.0329 | 0.0730 | 0.0108 |
| 1600 | 0.0818 | 0.0217 | 0.0494 | 0.0070 |
| 2000 | 0.0648 | 0.0203 | 0.0480 | 0.0064 |
| 2400 | 0.0504 | 0.0160 | 0.0373 | 0.0052 |
| **2800** | **0.0410** | **0.0143** | **0.0300** | **0.0040** |
| **Fold 4** | | | | |
| Step | Train Loss | Val Loss | WER | CER |
| 400 | 0.0653 | 0.0136 | 0.0364 | 0.0046 |
| 800 | 0.1008 | 0.0195 | 0.0442 | 0.0064 |
| 1200 | 0.0835 | 0.0167 | 0.0403 | 0.0055 |
| 1600 | 0.0641 | 0.0142 | 0.0325 | 0.0044 |
| 2000 | 0.0515 | 0.0113 | 0.0222 | 0.0031 |
| 2400 | 0.0426 | 0.0080 | 0.0181 | 0.0025 |
| **2800** | **0.0350** | **0.0065** | **0.0159** | **0.0023** |
| **Fold 5** | | | | |
| Step | Train Loss | Val Loss | WER | CER |
| 400 | 0.0519 | 0.0093 | 0.0228 | 0.0029 |
| 800 | 0.0876 | 0.0149 | 0.0407 | 0.0049 |
| 1200 | 0.0745 | 0.0104 | 0.0267 | 0.0036 |
| 1600 | 0.0584 | 0.0077 | 0.0209 | 0.0030 |
| 2000 | 0.0449 | 0.0070 | 0.0162 | 0.0023 |
| 2400 | 0.0361 | 0.0054 | 0.0156 | 0.0023 |
| **2800** | **0.0295** | **0.0046** | **0.0095** | **0.0014** |
