# Supplementary material for: Exploring Wav2Vec2 XLSR-53 and Whisper-small for Telugu language speech-to-text: a fine-tuning approach
Source: Front Artif Intell. 2026 Sep 9;9:1883049. doi: 10.3389/frai.2026.1883049 (PMC13597755; doi:10.3389/frai.2026.1883049)
Supplement: Supplementary file 5 [file Table_5.docx]

# SUPPLEMENTARY MATERIALS

**Table 5.** Performance analysis of Whisper-small for k = 2 across two folds on the k-fold validation folds.

| **Fold 1** | | | | |
| --- | --- | --- | --- | --- |
| Step | Train Loss | Val Loss | WER | CER |
| 400 | 0.5327 | 0.1547 | 0.4694 | 0.0978 |
| **800** | **0.0326** | **0.1639** | **0.3954** | **0.0775** |
| **Fold 2** | | | | |
| Step | Train Loss | Val Loss | WER | CER |
| 400 | 0.0842 | 0.0156 | 0.0677 | 0.0115 |
| **800** | **0.0133** | **0.0154** | **0.0752** | **0.0124** |
