# Supplementary material for: Exploring Wav2Vec2 XLSR-53 and Whisper-small for Telugu language speech-to-text: a fine-tuning approach
Source: Front Artif Intell. 2026 Sep 9;9:1883049. doi: 10.3389/frai.2026.1883049 (PMC13597755; doi:10.3389/frai.2026.1883049)
Supplement: Supplementary file 6 [file Table_6.docx]

# SUPPLEMENTARY MATERIALS

**Table 6.** Performance analysis of Whisper-small for k = 3 across three folds on the k-fold validation folds.

| **Fold 1** | | | | |
| --- | --- | --- | --- | --- |
| Step | Train Loss | Val Loss | WER | CER |
| 400 | 0.5378 | 0.1422 | 0.4630 | 0.0965 |
| 800 | 0.0474 | 0.1417 | 0.3761 | 0.0745 |
| **1200** | **0.0068** | **0.1608** | **0.3515** | **0.0681** |
| **Fold 2** | | | | |
| Step | Train Loss | Val Loss | WER | CER |
| 400 | 0.0477 | 0.0186 | 0.0937 | 0.0147 |
| 800 | 0.0143 | 0.0222 | 0.0968 | 0.0156 |
| **1200** | **0.0021** | **0.0176** | **0.0749** | **0.0121** |
| **Fold 3** | | | | |
| Step | Train Loss | Val Loss | WER | CER |
| 400 | 0.0070 | 0.0037 | 0.0194 | 0.0031 |
| 800 | 0.0066 | 0.0048 | 0.0237 | 0.0039 |
| **1200** | **0.0013** | **0.0024** | **0.0142** | **0.0021** |
