# Supplementary material for: Exploring Wav2Vec2 XLSR-53 and Whisper-small for Telugu language speech-to-text: a fine-tuning approach
Source: Front Artif Intell. 2026 Sep 9;9:1883049. doi: 10.3389/frai.2026.1883049 (PMC13597755; doi:10.3389/frai.2026.1883049)
Supplement: Supplementary file 7 [file Table_7.docx]

# SUPPLEMENTARY MATERIALS

**Table 7.** Performance analysis of Whisper-small for k = 4 across four folds on the k-fold validation folds.

| **Fold 1** | | | | |
| --- | --- | --- | --- | --- |
| Step | Train Loss | Val Loss | WER | CER |
| 400 | 0.536000 | 0.140445 | 0.452323 | 0.092056 |
| 800 | 0.055700 | 0.125187 | 0.374305 | 0.074552 |
| 1200 | 0.009800 | 0.143755 | 0.350967 | 0.065166 |
| **1600** | **0.001100** | **0.151775** | **0.328295** | **0.062121** |
| **Fold 2** | | | | |
| Step | Train Loss | Val Loss | WER | CER |
| 400 | 0.031900 | 0.007049 | 0.032294 | 0.005796 |
| 800 | 0.010600 | 0.009895 | 0.063019 | 0.010984 |
| 1200 | 0.002600 | 0.006829 | 0.043283 | 0.006661 |
| **1600** | **0.000300** | **0.005923** | **0.035210** | **0.005668** |
| **Fold 3** | | | | |
| Step | Train Loss | Val Loss | WER | CER |
| 400 | 0.003400 | 0.012776 | 0.090343 | 0.009887 |
| 800 | 0.007200 | 0.005324 | 0.030040 | 0.004738 |
| 1200 | 0.001500 | 0.003609 | 0.020249 | 0.003032 |
| **1600** | **0.000100** | **0.002999** | **0.013129** | **0.002085** |
| **Fold 4** | | | | |
| Step | Train Loss | Val Loss | WER | CER |
| 400 | 0.002900 | 0.005736 | 0.026756 | 0.004578 |
| 800 | 0.006000 | 0.004112 | 0.022074 | 0.003751 |
| 1200 | 0.001300 | 0.002291 | 0.008473 | 0.001685 |
| **1600** | **0.000100** | **0.001765** | **0.006912** | **0.001208** |
