# Supplementary material for: Exploring Wav2Vec2 XLSR-53 and Whisper-small for Telugu language speech-to-text: a fine-tuning approach
Source: Front Artif Intell. 2026 Sep 9;9:1883049. doi: 10.3389/frai.2026.1883049 (PMC13597755; doi:10.3389/frai.2026.1883049)
Supplement: Supplementary file 8 [file Table_8.docx]

# SUPPLEMENTARY MATERIALS

**Table 8.** Performance analysis of Whisper-small for k = 5 across five folds on the k-fold validation folds.

| **Fold 1** | | | | |
| --- | --- | --- | --- | --- |
| Step | Train Loss | Val Loss | WER | CER |
| 400 | 0.541500 | 0.136120 | 0.436182 | 0.089227 |
| 800 | 0.058300 | 0.123327 | 0.374029 | 0.073476 |
| 1200 | 0.012400 | 0.134373 | 0.339068 | 0.065562 |
| **1600** | **0.001600** | **0.146481** | **0.326304** | **0.060876** |
| **Fold 2** | | | | |
| Step | Train Loss | Val Loss | WER | CER |
| 400 | 0.024900 | 0.008294 | 0.035694 | 0.006044 |
| 800 | 0.010500 | 0.009221 | 0.051590 | 0.007818 |
| 1200 | 0.002700 | 0.006653 | 0.032627 | 0.004433 |
| **1600** | **0.000700** | **0.004493** | **0.022588** | **0.003345** |
| **Fold 3** | | | | |
| Step | Train Loss | Val Loss | WER | CER |
| 400 | 0.003200 | 0.008309 | 0.038472 | 0.006539 |
| 800 | 0.006500 | 0.005331 | 0.030329 | 0.004438 |
| 1200 | 0.002700 | 0.003317 | 0.017692 | 0.002893 |
| **1600** | **0.000300** | **0.002253** | **0.012918** | **0.002338** |
| **Fold 4** | | | | |
| Step | Train Loss | Val Loss | WER | CER |
| 400 | 0.002300 | 0.007152 | 0.040879 | 0.006394 |
| 800 | 0.006300 | 0.006153 | 0.033092 | 0.005242 |
| 1200 | 0.001300 | 0.002616 | 0.010567 | 0.001668 |
| **1600** | **0.000100** | **0.002003** | **0.008343** | **0.001112** |
| **Fold 5** | | | | |
| Step | Train Loss | Val Loss | WER | CER |
| 400 | 0.002200 | 0.004177 | 0.017827 | 0.002970 |
| 800 | 0.005500 | 0.002992 | 0.014206 | 0.002218 |
| 1200 | 0.001300 | 0.002525 | 0.010306 | 0.002099 |
| **1600** | **0.000100** | **0.002187** | **0.008635** | **0.001743** |
